# Evaluation of thermal conductivity models and dielectric properties in metal oxide-filled poly(butylene succinate-co-adipate) composites

**DOI:** 10.1038/s41598-024-64426-5

**Published:** 2024-06-13

**Authors:** Miks Bleija, Oskars Platnieks, Olesja Starkova, Jan Macutkevič, Dzmitry Tsyhanok, Liga Orlova, Sergejs Gaidukovs

**Affiliations:** 1 Institute of Chemistry and Chemical Technology, Faculty of Natural Sciences and Technology, Riga Technical University, P. Valdena 3, Riga, LV-1048 Latvia; 2 Institute for Mechanics of Materials, University of Latvia, Jelgavas 3, Riga, LV-1004 Latvia; 3 Faculty of Physics, Vilnius University, Sauletekio 9, 10222 Vilnius, Lithuania; 4 Institute of Materials and Surface Engineering, Faculty of Natural Sciences and Technology, Riga Technical University, P. Valdena 3, Riga, LV-1048 Latvia

**Keywords:** Polymers, Electronic properties and materials, Nanoparticles, Electronic properties and materials, Nanoparticles, Nanocomposites, Engineering

## Abstract

This study examines how various nanofillers impact thermal conductivity, dielectric characteristics, and electromagnetic interference (EMI) shielding potential of bio-based and biodegradable poly(butylene succinate-co-adipate) (PBSA). TiO_2_, NiFe_2_O_4_, Fe_2_O_3_, and Fe_3_O_4_ were selected as fillers for nanocomposites at 4–50 vol.% (12–81 wt.%). The nanocomposites were analyzed in three domains: structural (scanning electron microscopy, energy dispersive X-ray spectroscopy mapping, density, tensile testing), thermal (light flash analysis, literature models), and dielectric (AC conductivity, permittivity, EM shielding effectiveness (SE)). The investigated fillers showed good dispersion and compatibility with the PBSA matrix. LFA was analyzed according to literature models, where Bruggeman and Agari models showed the best fit at high concentrations. The dielectric analysis revealed that most of the nanocomposites did not reach percolation; thus, producing thermally conductive plastics that are electrically insulating. EMI shielding was limited to frequencies below 10 Hz, with the notable exception of Fe_3_O_4_ (100 nm and loading of > 25 vol.%), which showed shielding at frequencies up to 10^5^ Hz. The investigated composites based on a biodegradable polyester and abundant metal oxide nanofillers are suitable for the production of cheap, ecological, and electrically insulating heat dissipation solutions required for modern and lightweight applications.

## Introduction

In the dynamic and rapidly evolving landscape of modern technology, particularly in the context of new advances in microelectronics, 5G communication equipment, lightweight aerospace, and electric transportation technologies, there has been a significant drive towards higher frequency, power, and transistor density in electronic systems and components. The trend towards miniaturization and growing power densities, as well as a push for energy-efficiency in electronic devices, requires new solutions for efficient heat dissipation for long-term operation^1^. A broad understanding of thermally conductive materials is necessary to achieve the desired goal, which requires engineering parameters, models, and their verification.

The development of thermally conductive materials that are electrically insulating, such as polymer composites, has become increasingly important in materials science^2^. Thermally conductive insulators find use in heat dissipation applications where electrical conductivity might cause short-circuits, electromagnetic non-compliance, or other undesirable effects^3–5^. In the applications where electrical conductivity is permissible, conductive fillers can impart composites with electromagnetic (EM) shielding, protecting against electromagnetic interference (EMI) and electrical noise, thus improving the operational stability of devices^6^.

Polymers, traditionally favored for their excellent flexibility, low density, corrosion resistance, and energy-saving low-temperature processability, encounter limitations due to their inherently low dielectric constants and thermal conductivity. This has led to a surge in interest in organic–inorganic hybrid composite strategies, particularly focusing on the integration of thermally conductive fillers in the polymer matrix. The literature shows that for applications focused on heat dissipation, a mix of fossil-based commodity and engineering plastics have been studied, e.g., polypropylene^7^, polyamide-6^8^, low density polyethylene^9^, polyvinylidene fluoride^10^. New emerging bio-based and biodegradable matrix solutions have been underrepresented in the thermally conductive composite literature. Lule et al. reported the use of 5 wt.% surface modified AlN in a poly(butylene succinate) (PBS) matrix, resulting in a relatively low thermal conductivity of up to 0.32 Wm^−1^ K^−1^^11^. Another study from the same group used 18.6 vol.% surface treated Si_3_N_4_ to reach a thermal conductivity of 0.71 Wm^−1^ K^−1^^12^. Liu et al. developed a complex directionally oriented composite of PBS, 3 vol.% carbon nanotubes, and 20 vol.% boron nitride, which had an in-axis thermal conductivity of 2.2 Wm^−1^ K^−1^^13^. Our previous study of carbon nanotube/iron oxide hybrids in a PBS matrix showed thermal conductivity of up to 0.30 Wm^−1^ K^−1^^14^.

Present efforts to enhance the thermal conductivity of polymers are concentrated on the selective incorporation of mostly cheap nanofillers known for their high thermal conductivity. Commonly, metal oxide, ceramic, and carbon-based fillers are used for the preparation of thermally conductive polymer composites^15^. Particularly, carbon-based fillers are more efficient in the formation of electrically conductive networks, which eliminates the insulating properties of the polymer matrix. In addition to being quite expensive, carbon-based fillers with large aspect ratios introduce additional challenges in viscosity, dispersion, and agglomeration during the preparation stages of the nanocomposite^15^. Ceramic fillers used in the literature mainly include various nitrides, which have several challenges associated with them, like processability and anisotropic heat dissipation in the 3D structure, as well as their cost^16^.

Recent studies on the innovative application of metal oxide-based fillers designed to enhance thermal conductivity and heat dissipation are scarce. Research is mostly confined to non-degradable plastics. Weidenfeller et al. investigated polypropylene composites filled with various metal and metal oxide fillers and found particle geometry and thus interconnectivity to be very important in achieving high thermal conductivity values^17^. Fu et al.^18^ used several commonly available thermally conductive fillers to enhance the thermal conductivity of epoxy adhesives, achieving improvements of 200–300% at high filler loadings while also remarking on the importance of filler geometry. Sahu et al.^19^ investigated microscale TiO_2_ and epoxy composite thermal conductivities and their correlation with models from the literature. They found a thermal percolation threshold at around 17.5–20.0 vol.% and an increase from 0.35 to 1.1 Wm^-1^ K^-1^, which disagreed with the theoretical models but is likely related to filler orientation caused by processing difficulties.

**Figure 1:**
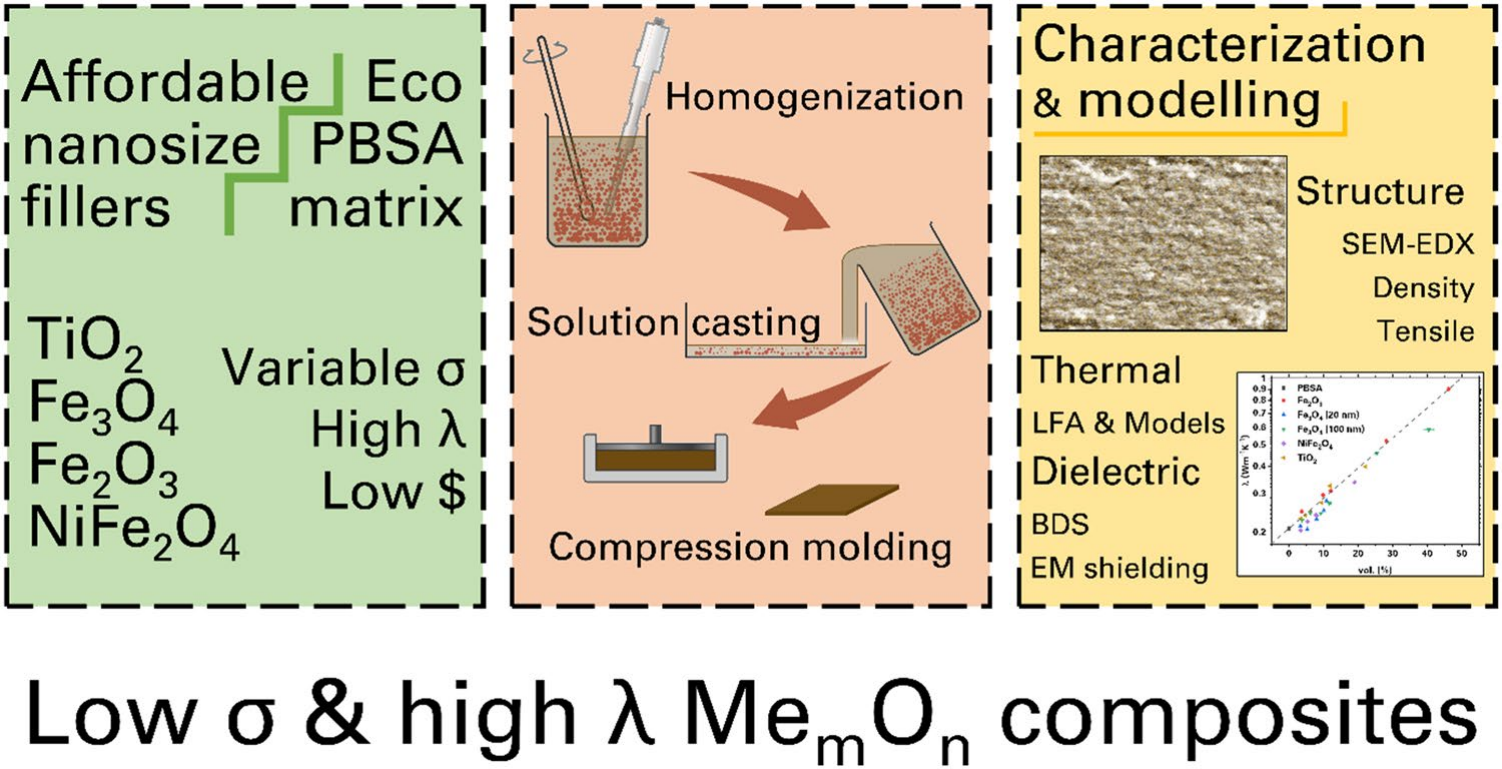
Schematic design methodology of this work.

In this investigation, we assess the effects of various metal oxide fillers, dispersed in a compostable polyester matrix, on thermal conductivity, tensile strength, dielectric properties, and electromagnetic interference (EMI) shielding, as depicted in Fig. 1. The bio-based, biodegradable polymer poly(butylene succinate-co-adipate) (PBSA) matrix was selected for its low melting point (reduced processing costs), exceptional ductility, and compatibility with a wide range of processing methods. This study aims to compare the impacts of different fillers, examining the influence of filler loading and particle size. Additionally, we analyze the experimental data in relation to compliance with and deviations from four distinct thermal conductivity models, thereby highlighting the interaction between the fillers and the PBSA matrix in enhancing the thermal properties of the material.

## Materials and methods

### Materials

Poly(butylene succinate-co-adipate) (PBSA) was selected as the composite matrix. PBSA grade FD92PM was purchased from PTT MCC Biochem Co., Ltd., (Bangkok, Thailand). It is a semicrystalline and biodegradable thermoplastic polyester in granule form, mainly used for blown and extruded films in packaging. Metal oxides were chosen as composite fillers. α-Fe_2_O_3_ (hematite, 99%, APS 100 nm), Fe_3_O_4_ (99%, APS 100 nm), NiFe_2_O_4_ (98%, APS 30 nm^20^), and β-TiO_2_ (anatase, 99.5%, APS 5 nm) were purchased from US Research Nanomaterials, Inc. (Houston, TX, USA). Fe_3_O_4_ (99.5%, APS 20 nm^21^) was purchased from Oocap, Inc. (Las Cruces, NM, USA). Chloroform (CAS No.: 67-66-3) (≥ 99%), which was used in the solvent casting process, was purchased from Merck KGaA (Darmstadt, Germany). The PBSA was dried under vacuum (70 °C, 24 h) (J.P Selecta, Barcelona, Spain) before use as per manufacturer recommendations. The nanofillers were used as received, without any further processing.

### Composite preparation

The composites were prepared using a simple solvent-based method. First, the fillers were dispersed in chloroform using sonication for 15 min (Hielscher UIS250V, Hielscher Ultrasonics GmbH, Teltow, Germany). Afterwards, the nanoparticle suspension was combined with a low concentration solution of PBSA in chloroform, and homogenized via sonication (15 min), followed by high-shear mixing at 10000 RPM for 30 min (Silverson L5M-A, Silverson Machines Ltd., Chesham, United Kingdom). The polymer-nanoparticle solution was then cast into a Petri dish and left overnight in a fume hood. Finally, the polymer film was further desiccated in a vacuum drying oven for 24 h at 70 °C. The filler loading values were chosen according to experiment design, to find critical values and evaluate filler dispersion and distribution (Table 1). The solvent cast composites were formed into 0.7 and 0.45 mm thick plates using compression molding at 135 °C for 3 min (Carver Inc., Wabash, IN, USA) followed by rapid cooling between steel plates at room temperature. Some filler was lost as particles separated from the cast polymer films. To ensure adequate analysis, filler volume percentages were recalculated by extrapolation from measured density values. To reflect the real filler percentage, samples were abbreviated from extrapolated vol.% (Table 1). Some of the highly loaded composites were too brittle for processing samples to precise shapes required for the specific measurements.

**Table 1:** Prepared PBSA nanocomposites with experimental and theoretical density values, and extrapolated filler concentrations.

Nos.	Filler	vol (%)	wt (%)	${\rho }_{EX}$ (g·cm^−3^)	${\rho }_{th}$ (g·cm-^3^)	V_p_ (%)	vol_EX_ (%)	Abbreviated vol (%)
1	PBSA	–	–	1.227 ± 0.001	1.227	–	–	–
2	TiO_2_ (5 nm)	4.2	12.3%	1.318 ± 0.003	1.340	1.6	3.40 ± 0.01	3
3		7.4	20.4%	1.356 ± 0.012	1.426	4.9	4.81 ± 0.04	5
4		12.1	30.3%	1.481 ± 0.012	1.549	4.4	9.51 ± 0.08	10
5		15.2	36.3%	1.545 ± 0.034	1.634	5.4	11.88 ± 0.26	12
6		30.0	57.7%	1.820 ± 0.010	2.029	10.3	22.17 ± 0.13	22
7	NiFe_2_O_4_ (30 nm)	4.0	15.5%	1.370 ± 0.015	1.393	1.7	3.44 ± 0.04	3
8		6.8	24.3%	1.452 ± 0.038	1.511	3.9	5.44 ± 0.14	5
9		10.5	34.0%	1.562 ± 0.010	1.664	6.1	8.08 ± 0.05	8
10		12.9	39.3%	1.554 ± 0.013	1.761	11.7	7.90 ± 0.07	8*
11		30.0	65.2%	2.015 ± 0.024	2.470	18.4	19.02 ± 0.22	19
12		50.0	81.4%	2.624 ± 0.010	3.298	20.4	33.72 ± 0.13	34
13	Fe_2_O_3_ (100 nm)	4.0	15.2%	1.377 ± 0.009	1.389	0.9	3.73 ± 0.03	4
14		6.9	24.0%	1.479 ± 0.029	1.504	1.7	6.27 ± 0.12	6
15		10.7	33.7%	1.626 ± 0.008	1.655	1.7	9.94 ± 0.05	10
16		13.0	39.0%	1.714 ± 0.021	1.751	2.1	12.12 ± 0.15	12
17		30.0	64.7%	2.359 ± 0.010	2.432	3.0	28.20 ± 0.12	28
18		50.0	81.0%	3.083 ± 0.006	3.234	4.6	46.26 ± 0.08	46
19	Fe_3_O_4_ (100 nm)	4.0	14.9%	1.375 ± 0.012	1.384	0.6	3.81 ± 0.03	4
20		6.9	23.7%	1.467 ± 0.010	1.496	1.9	6.20 ± 0.04	6
21		10.8	33.5%	1.583 ± 0.022	1.646	3.8	9.20 ± 0.13	9
22		13.2	38.8%	1.678 ± 0.083	1.740	3.5	11.65 ± 0.58	12
23		30.0	64.1%	2.214 ± 0.027	2.389	7.4	25.47 ± 0.31	25
24		50.0	80.6%	2.793 ± 0.106	3.164	11.7	40.43 ± 1.53	40
25	Fe_3_O_4_ (20 nm)	4.0	14.9%	1.356 ± 0.006	1.384	2.0	3.34 ± 0.01	3
26		6.9	23.6%	1.433 ± 0.024	1.495	4.1	5.32 ± 0.09	5
27		10.8	33.4%	1.539 ± 0.003	1.645	6.4	8.06 ± 0.02	8
28		13.2	38.8%	1.619 ± 0.015	1.739	6.9	10.11 ± 0.09	10
29		30.0	64.0%	1.227 ± 0.001	2.389	31.0	10.89 ± 0.28	11

### Characterization

- Structure characterization

The nanocomposite density was measured using hydrostatic weighing at room temperature. The sample weight was measured in air and in ethanol at room temperature on analytical scales Sartorius KBBA 100 with a YDK 01 density measurement kit (Sartorius AG, Göttingen, Germany), and the sample density was calculated according to the equation specified by the manufacturer:

1 $${\rho }_{EX}=\frac{{W}_{a}\left({\rho }_{\text{fl}}-{\rho }_{a}\right)}{0.99983\left({W}_{\text{a}}- {W}_{\text{fl}}\right)} +{\rho }_{a}$$

where ${W}_{a}$ (g) is the sample’s weight in air, ${W}_{\text{fl}}$ is the sample’s weight measured submerged in ethanol (g), ${\rho }_{\text{fl}}$ is the density of used ethanol (0.805 g∙cm^−3^), which was determined with a hydrometer, and ${\rho }_{a}$ is the density of air (0.0012 g∙cm^−3^).

Scanning electron miscroscopy (SEM) and energy-dispersive X-ray spectroscopy (EDX) mapping were carried out on cryofractured (liquid N_2_) Fe_2_O_3_ (4, 28, 46 vol.%) and Fe_3_O_4_ (100 nm) (4, 25, 40 vol.%) samples. FEI Nova NanoSEM 650 Schottky field emission scanning electron microscope (FESEM, Hillsboro, OR, USA) was used at a voltage of 10 kV to examine the fracture surface morphology, without any additional surface coatings.

Tensile testing was carried out at room temperature using a universal testing machine, the Tinius Olsen 25ST (Horsham, PA, USA), equipped with a 5 kN load cell at a speed of 1 mm per minute up to 2% strain, followed by 2 mm per minute until specimen fracture. The dog-bone shaped specimens with a gauge length of 21 mm, a width of 5 mm, and a thickness of 0.4 mm were cut out of compression-molded composite films.

- Thermal characterization

The thermal dissipative properties of the composites were obtained using light flash analysis apparatus LFA 447 NanoFlash (NETZSCH-Gerätebau GmbH, Selb, Germany) equipped with a standard 12.7 mm sample holder for through-plane diffusivity measurements according to ISO 22007-4. Square samples were coated with a graphite-based coating Graphit 33 as per manufacturer recommendations, to ensure equal absorbance and comparability with the reference samples. The thermal conductivity is calculated according to the equation:

2 $$\lambda \left(T\right)=\alpha \left(T\right)\rho {\left(T\right)C}_{p}\left(T\right)$$

where $\lambda$ is thermal conductivity (Wm^−1^ K^−1^), $\alpha$ is diffusivity (mm^2^s^−1^), $\rho$ is density (g∙cm^−3^) ${C}_{p}$ is specific heat capacity (Jg^−1^ K^−1^).

LFA measures sample diffusivity according to the half-time method:

3 $$\alpha =\frac{0.1388{d}^{2}}{{t}_\frac{1}{2}}$$

where d is the sample thickness, and ${t}_\frac{1}{2}$ is the half-rise time of the IR detector signal. As polymer composites are too insulating to use the adiabatic equation, the Cowan model^22^ provided by the LFA analysis software suite was determined as the most accurate. Sample specific heat capacities were determined using the LFA analysis software suite by comparison to a reference with a known heat capacity. As $\lambda$ is temperature dependent, measurements were carried out at three temperatures: 25, 35, 45 °C. Each sample was subjected to 5 consecutive measurements with a 120 s delay to allow the samples to return to thermal equilibrium. The measurement standard deviation is within 0.02 Wm^−1^ K^-1^.

To model the thermal conductivity of the composites, we applied several models from the literature (Table 3).

To determine the thermal activation energy or the thermal dependence of thermal conductivity, a modified Arrhenius equation^23^ was used:

4 $$\lambda ={\lambda }_{0}{e}^{\left(-\frac{{E}_{a}}{kT}\right)}$$

where ${\lambda }_{0}$ is the extrapolated inherent thermal conductivity at infinite temperature (Wm^−1^ K^−1^), ${E}_{a}$ is the energy of thermal activation, $T$ is absolute temperature (K), and $k$ is the Boltzmann constant (8.617∙10^−5^ eVK^−1^). In this case ${E}_{a}$ is calculated by taking the slope of the Arrhenius plot. It must be noted that thermal conductivity activation energies are valid only within the bounds of the tested temperature interval.

- Electric-dielectric characterization

The electric and dielectric properties—AC conductivity and dielectric permittivity—were characterized using a broadband dielectric spectroscope Novocontrol BDS 50 (Novocontrol Technologies GmbH and Co. KG, Montabaur, Germany). Composites were cut into 30 mm disks and placed between plate electrodes of the device. The measurements were carried out at frequencies from 10^−2^ to 4 × 10^7^ Hz at room temperature.

Dielectric EMI shielding efficiency was calculated over the tested frequency from the real and imaginary dielectric components using the following equations ^24^:

5 $$\Gamma =\frac{\sqrt{\frac{\left({\mu }{\prime}-j{\mu }^{{\prime}{\prime}}\right)}{\left({\varepsilon }{\prime}-j{\varepsilon }^{{\prime}{\prime}}\right)}}\text{tanh}\left(j\frac{2\pi fd}{c}\sqrt{\left({\mu }{\prime}-j{\mu }^{{\prime}{\prime}}\right)\left({\varepsilon }{\prime}-j{\varepsilon }^{{\prime}{\prime}}\right)}\right)-1}{\sqrt{\frac{\left({\mu }{\prime}-j{\mu }^{{\prime}{\prime}}\right)}{\left({\varepsilon }{\prime}-j{\varepsilon }^{{\prime}{\prime}}\right)}}\text{tanh}\left(j\frac{2\pi fd}{c}\sqrt{\left({\mu }{\prime}-j{\mu }^{{\prime}{\prime}}\right)\left({\varepsilon }{\prime}-j{\varepsilon }^{{\prime}{\prime}}\right)}\right)+1}$$

6 $$T={e}^{-\left(j\frac{2\pi fd}{c}\sqrt{\left({\mu }{\prime}-j{\mu }^{{\prime}{\prime}}\right)\left({\varepsilon }{\prime}-j{\varepsilon }^{{\prime}{\prime}}\right)}\right)}$$

7 $$SE=-20\text{log}\left|\frac{\left(1-{\Gamma }^{2}\right)T}{1-{T}^{2}{\Gamma }^{2}}\right|$$

where $\Gamma$ is the reflectance, $T$ is the transmittance, $f$ is the frequency, $d$ is the sample thickness, $c$ is the speed of light, $\left({\varepsilon }{\prime}-j{\varepsilon }^{{\prime}{\prime}}\right)$ is the complex permittivity, $\left({\mu }{\prime}-j{\mu }^{{\prime}{\prime}}\right)$ is the complex permeability (taken to be 1 when evaluating only dielectric effects), $SE$ is the shielding efficiency (dB).

In the microwave frequency range from 25 to 40 GHz, a custom-made (Faculty of Physics, University of Vilnius, Lithuania) thin rod waveguide spectrometer as described in the reference^25^ was used. Disc-shaped compression-molded specimens with a thickness of 0.45 mm were placed in the waveguide holder. The electromagnetic shielding properties of the nanocomposites in the tested range were calculated according to equations^26,27^:

8 $$\left\{\begin{array}{c}{S}_{11}=\frac{-j\left[{\left(\frac{{k}_{z}}{{k}_{2z}}\right)}^{2}-1\right]\text{sin}({k}_{2z}\tau )}{2j\left(\frac{{k}_{z}}{{k}_{2z}}\right)\text{cos}\left({k}_{2z}\tau \right)+\left[{\left(\frac{{k}_{z}}{{k}_{2z}}\right)}^{2}+1\right]\text{sin}({k}_{2z}\tau )}\\ {S}_{21}=\frac{2\left(\frac{{k}_{2z}}{{k}_{z}}\right)}{-2\left(\frac{{k}_{2z}}{{k}_{z}}\right)\text{cos}\left({k}_{2z}\tau \right)+j\left[{\frac{{k}_{2z}}{{k}_{z}}}^{2}+1\right]\text{sin}({k}_{2z}\tau )}\end{array}\right.$$

where ${k}_{z}=\frac{2\pi }{\lambda }$ and ${k}_{2z}=\frac{2\pi {\varepsilon }^{2}}{\lambda }$ are wave numbers in the vacuum and the sample’s media correspondingly, and $\tau$ is the thickness of the layer. The absorption of the layer was calculated according to:

9 $$A=1-{\left({S}_{11}\right)}^{2}-{\left({S}_{21}\right)}^{2}$$

## Results and discussion

### Structure and morphology

During the processing stage, some filler was lost as particles separated from the cast polymer films. As a result, it became necessary to conduct density measurements to ascertain the volume concentration of filler that remained. True volume fraction was extrapolated from the density measurements (${\rho }_{EX}$) and rule-of-mixtures (${\rho }_{th}$) assuming that the samples have no voids present in the structure. The composites filled with 100 nm particles exhibited the smallest volumetric deviation (${V}_{p}=\frac{{\rho }_{th}}{{\rho }_{EX}}\%$), with the extent of this deviation varying according to the average particle size. For fillers with a size below 50 nm, the non-magnetic TiO_2_ displayed the least deviation. Considering both aspects, we suggest that the compatibility between the filler and the matrix in our composite is influenced by two main factors. Firstly, larger particles possess a greater surface area, which allows for the creation of a higher number of adhesive bonds. Secondly, during the solvent casting process, small magnetic particles tend to stick together weakly through dipole–dipole interactions, which in turn hinders their ability to adhere to the matrix. The surface energy of nanoparticles also varies with particle size^28^. However, this is unlikely to significantly affect the wettability of the filler, due to the relatively large size of our particles.

**Figure 2:**
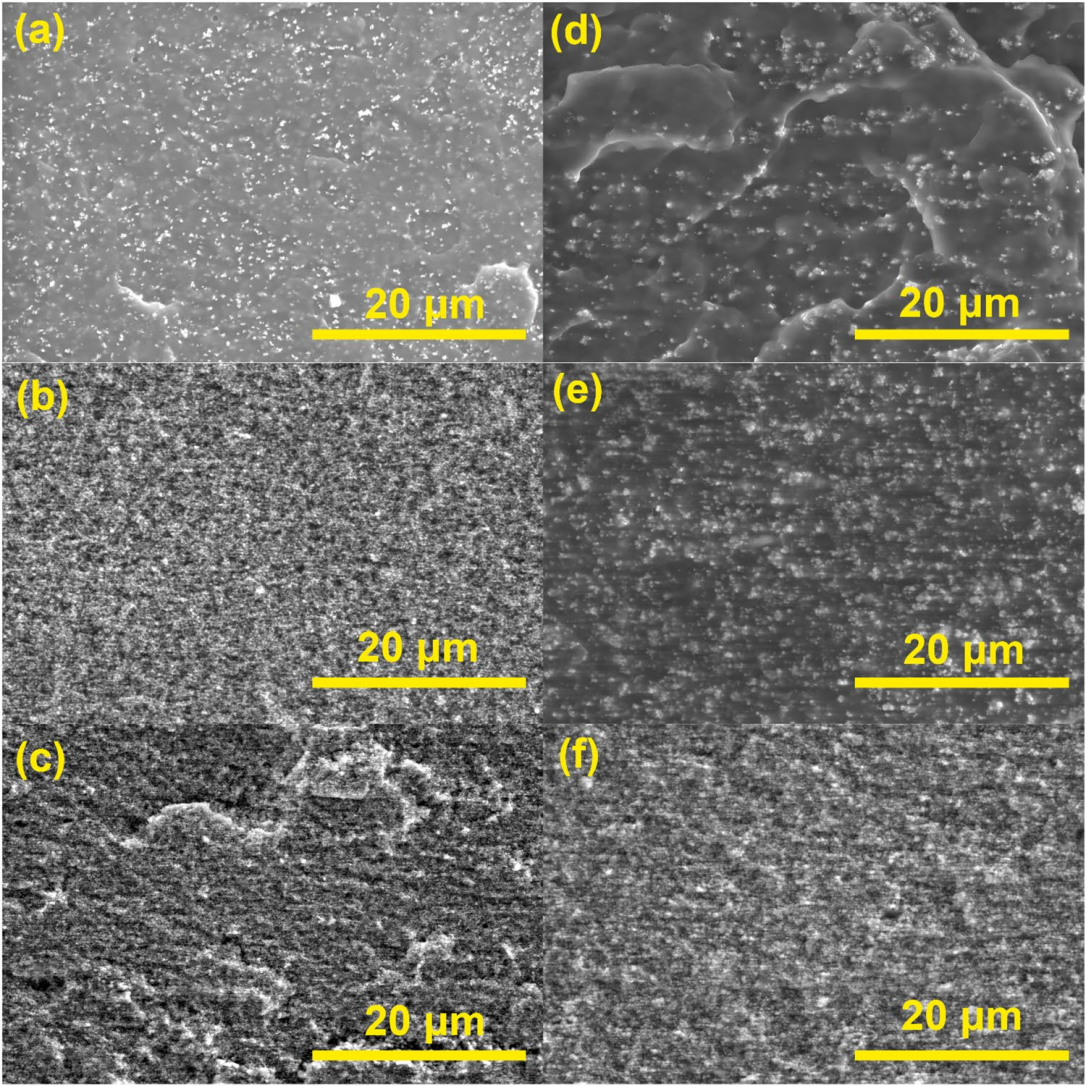
Fe_2_O_3_ composites at (**a**) 4 vol.%, (**b**) 28 vol.%, (**c**) 46 vol.%, and Fe_3_O_4_ (100 nm) composites at (**d**) 4 vol.%, (**e**) 25 vol.%, (**f**) 40 vol.% at 2 500 × magnification.

The dispersion and distribution of nanofillers in composites filled with Fe_2_O_3_ and Fe_3_O_4_ (100 nm) were qualitatively evaluated using SEM (Fig. 2). This assessment focused on Fe_2_O_3_ and Fe_3_O_4_ (100 nm) composites with filler concentrations of 4, 28/25, and 46/40 vol.%, which have the highest thermal conductivities among selected fillers. At the lowest concentrations, Fe_3_O_4_ particles tended to agglomerate in uniform clusters with a size under 1 μm (Fig. 2d). In contrast, Fe_2_O_3_ showed better dispersion and smaller clusters or no agglomeration (Fig. 2a). Overall, both fillers show uniform distribution and good dispersion within the composite. As shown in our previous study^14^, Fe_3_O_4_ particles are likely to re-agglomerate due to magnetic interaction after sonication and homogenization. This impacts particle dispersion and promotes formation of clusters^29^. The clusters are small enough to not get destroyed during compression molding.

With an increase in the filler concentration (28 vol.%), Fe_2_O_3_ composite had a much more densely packed structure with filler packed in small clusters (Fig. 2b). These clusters are also visible at fracture surface, showing pull-outs during crack formation. The size of the pulled-out holes represents the formed cluster sizes. This morphology resulted in a relatively brittle composite structure. For Fe_3_O_4_ the distribution is less regular in both the micro and the macro scales (Fig. 2e), but notable is the lack of separation between matrix and filler. This indicates that Fe_3_O_4_ has a good compatibility with PBSA with used processing methods. Nanofillers are more likely to agglomerate at higher concentrations which can further impact polymer crystallinity and molecular mobility^30^. The strong agglomeration of nanoparticles is visible for both highly loaded compositions (Fig. 2c and f) as large pulled-out cavities. It should be noted that both compositions reached a very high packing density. Micrographs at magnifications (1 000–10 000 ×) are available in the supplemental material (Figures S1–S10).

**Figure 3:**
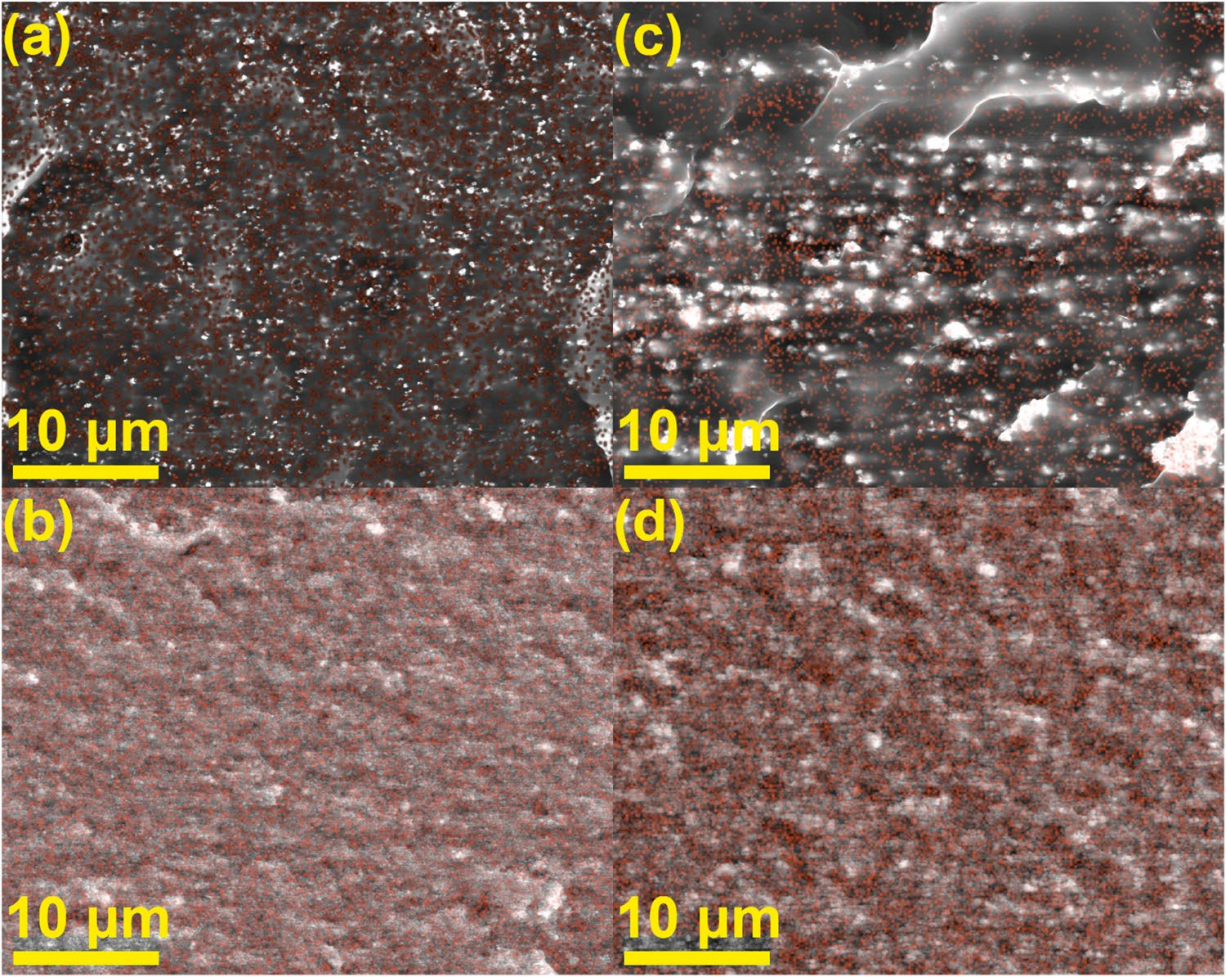
SEM–EDS element mapping for Fe_2_O_3_/PBSA composites at (**a**) 4 vol.%, (**b**) 46 vol.%, and for Fe_3_O_4_ (100 nm)/PBSA composites at (**c**) 4 vol.%, (**d**) 40 vol.% showing the distribution of Fe (red).

To complement SEM results and show the exceptional distribution of nanoparticles SEM–EDS mapping was performed (Fig. 3). The most notable observation is that at 4 vol.% loading nanoparticles show almost no agglomeration (Fig. 3a and c). At a high loading the polymer matrix still retains a continuous network and effectively covers nanoparticles in the composite structure, even with increased filler agglomeration.

**Figure 4:**
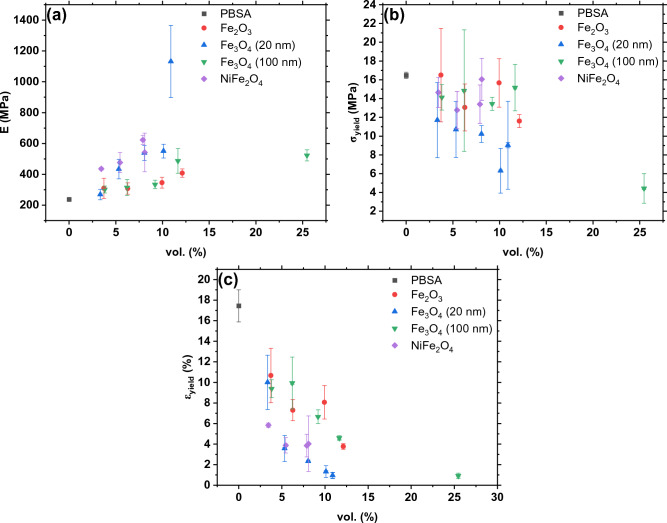
Tensile properties of PBSA/oxide nanoparticle composites: (**a**) elastic modulus, (**b**) yield strength, and (**c**) yield strain.

Figure 4 illustrates the tensile properties of the nanocomposites as a function of filler volume concentration: elastic modulus (Fig. 4a), yield strength (Fig. 4b), equivalent to ultimate stress for our samples, and yield strain (Fig. 4c). The introduction of fillers elevated the elastic modulus of the composites (Fig. 4a), reaching a peak at 1132 ± 234 MPa for 11 vol.% Fe_3_O_4_ (20 nm). With increasing filler content, the composites transition to a more elastic yet brittle state. The NiFe_2_O_4_ and Fe_3_O_4_ (20 nm) fillers showed the most consistent and notable enhancement of elastic modulus. The yield strength (Fig. 4b) of the composites remained at the level of the neat system for most fillers with loadings up to around 10 vol.%. For Fe_3_O_4_ (20 nm) a more pronounced drop in yield strength was observed.

As described previously^31^, PBSA undergoes a completely plastic failure characterized by extensive necking post-yield due to the orientation of the polymer chains; fractures occur at exceedingly high strains (elongation at break up to 250%). This orientation effect is maintained solely in composites with minimal filler concentrations, exemplified by the elongation at break values of 106.13 ± 59.39% for 4 vol.% Fe_2_O_3_, and 138.25 ± 16.93% and 81.70 ± 11.44% for 4 and 6 vol.% Fe_3_O_4_ 100 nm, respectively. Particle agglomeration serves to create stress concentrators, thereby reducing the overall ultimate strain by promoting crack initiation and facilitating crack propagation^32^. The agglomeration is related to filler characteristics, concentration, size, and dispersion. The likelihood of agglomeration and consequent embrittlement correlates with the average particle size of the fillers; composites filled with TiO_2_ (5 nm) were excessively brittle to be molded into dog-bone-shaped specimens at higher filler loadings. This is corroborated by the reduced yield strains observed in NiFe_2_O_4_ and Fe_3_O_4_ (20 nm) composites (Fig. 4c). Beyond the yield point (Fig. 4c), all composites, with the exception of those previously mentioned, fractured at the onset of necking.

### Thermal conductivity evaluation and temperature dependence

**Figure 5:**
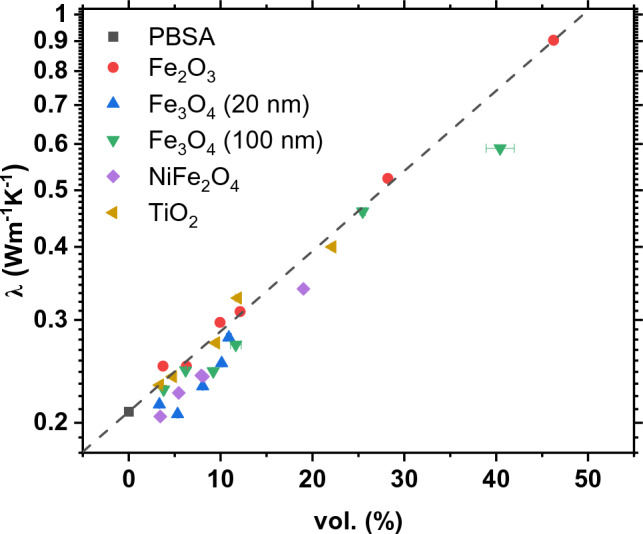
Composite thermal conductivity dependence on filler volume at 25 °C.

Figure 5 shows the thermal conductivity of composites at 25 °C in a semi-log graph. The neat PBSA had a conductivity of 0.209 Wm^−1^ K^−1^, while the introduction of fillers increased the values in line with an exponential proportionality $\lambda \propto \left({\lambda }_{m}\cdot \text{exp}\left({V}_{f}\sqrt{0.001}\right)\right)$. The highest conductivities were achieved with composites containing the highest concentrations of Fe_2_O_3_ and Fe_3_O_4_ nanoparticles (100 nm). This is attributed to an increase in connectivity of filler particles as the filler content rises. At intermediate filler concentrations, Fe_2_O_3_ and TiO_2_ showed superior volume-specific conductivities, whereas NiFe_2_O_4_ and 20 nm Fe_3_O_4_ particles had little to no conductivity improvement. The difference in filler conductivities ${\lambda }_{f}$ and the matrix conductivity ${\lambda }_{m}$, has a negligible effect on the composite conductivities at low interconnectivities of filler particles. The geometry and size of the particles can also affect conductivity, by introducing lower-conductivity interfaces within the composites due to a difference in the surface area. It is expected that larger particles would have a higher influence on the thermal conductivity, however, in this case TiO_2_ particles are an order of magnitude smaller than Fe_2_O_3_ particles, yet they exhibit a similar effect on the thermal properties. Table 2 provides a comparative analysis of various thermally conductive composites, detailing similar filler concentrations reported in the literature alongside the composites investigated in this work.

**Table 2:** Overview of various thermally conductive polymer composites documented in the literature.

Filler	Matrix	Loading	λ, (Wm^-1^ K^-1^)	Refs.
AlN	PBS	5 wt.%	0.32	^11^
Surface treated Si_3_N_4_	PBS	18.6 vol.%	0.71	^12^
BN	PDMS	30 vol.%	0.5–0.8	^33^
Al_2_O_3_	HDPE	30 vol.%	0.4–0.5	^34^
Fe_2_O_3_	PBSA	28 vol.%	0.524	This work
		46 vol.%	0.903	
Fe_2_O_3_	Acrylic copolymer	25 wt.%	~ 0.3	^35^
		45 wt.%	~ 0.7	
Fe_3_O_4_ (100 nm)	PBSA	25 vol.%	0.471	This work
		40 vol.%	0.59	
Fe_3_O_4_	PP	30 vol.%	~ 0.8	^17^
Fe_3_O_4_	Paraffin	10 wt.%	0.31–0.43	^36^
		20 wt.%	0.33–0.47	
NiFe_2_O_4_	PBSA	19 vol.%	0.339	This work
ZnFe_2_O_4_	ABS	14 wt.%	~ 0.21	^37^
Silanised MgFe_2_O_4_	PDHS	37 wt.%	1.33	^38^
TiO_2_	PBSA	22 vol.%	0.4	This work
TiO_2_	PP	25 vol.%	~ 0.4	^39^

To evaluate how well certain mathematical models can predict the heat-related properties of composite materials, we used a variety of models represented in the literature. These models were applied to data that we collected at a temperature of 25 °C. Table 3 provides a detailed presentation of the mathematical equations for the models.

**Table 3:** Models used for thermal conductivity fitting.

Model	Equation	Refs.
Maxwell	$\frac{{\lambda }_{c}}{{\lambda }_{m}}=\frac{{\lambda }_{f}+2{\lambda }_{m}+2{V}_{f}\left({\lambda }_{f}-{\lambda }_{m}\right)}{{\lambda }_{f}+2{\lambda }_{m}-{V}_{f}\left({\lambda }_{f}-{\lambda }_{m}\right)}$	^40–43^
Hatta	$\frac{{\lambda }_{c}}{{\lambda }_{m}}=1+\frac{{V}_{f}}{\frac{1}{3}\left(1-{V}_{f}\right)+\frac{{\lambda }_{m}}{{\lambda }_{f}-{\lambda }_{m}}}$	^44^
Bruggeman	$1-{V}_{f}=\left(\frac{{\lambda }_{f}-{\lambda }_{c}}{{\lambda }_{f}-{\lambda }_{m}}\right){\left(\frac{{\lambda }_{m}}{{\lambda }_{c}}\right)}^\frac{1}{3}$	^40,42,43,45^
Lewis-Nielsen	$\frac{{\lambda }_{c}}{{\lambda }_{m}}=\frac{1+A\cdot B\cdot {V}_{f}}{1-B\cdot\Phi \cdot {V}_{f}}$ $B=\frac{\frac{{\lambda }_{f}}{{\lambda }_{m}}-1}{\frac{{\lambda }_{f}}{{\lambda }_{m}}+A}$ $A={k}_{E}-1$ $\Phi =1+\left(\frac{1-{V}_{fmax}}{{{V}_{fmax}}^{2}}\right){\cdot V}_{f}$	^43,45,46^
Agari	$\text{log}\left({\lambda }_{c}\right)={V}_{f}{C}_{2}\text{log}\left({\lambda }_{f}\right)+\left(1-{V}_{f}\right)\text{log}({C}_{1}{\lambda }_{m})$	^40–42,46^

The Maxwell model is one of the most used models^40–43^, also known as Maxwell-Garnett^40^ and Maxwell-Eucken^41^, it parallels the Hashin-Strickman lower bounds and is prominent in describing thermal conductivity in randomly dispersed spherical non-interacting particles in a continuous polymer matrix. It assumes a continuous temperature profile at the filler surface, which proves effective at a macroscopic level. It becomes inadequate at significantly smaller nanoscales, where the assumption fails because thermal energy carriers, whether electrons or phonons, are scattered at the interface^40^. The Hatta model^44^, based on Eshelby’s modified equivalent inclusion model model^47^, is mathematically equivalent to the Maxwell model. However, since their fitting curves coincide, only the Maxwell model is considered in further analysis.

The Bruggeman model was developed using different assumptions for permeability and field strength compared to the Maxwell model^40,43^. The model assumes that a composite material can be constructed incrementally by introducing infinitesimal changes to an already existing material. Thus, this approach is also called the differential effective medium theory. The Bruggeman model offers an advantage by taking into account interactions among randomly distributed fillers, and it can be extended to multi-component systems^45^. It also provides highly accurate predictions for composites with high volume fractions of fillers.

The Lewis-Nielsen model is a semi-empirical model that considers the geometric features (shape, orientation and degree of packing) of filler particles^43,45,46^. Two additional parameters are introduced to the model: $A$—a constant related to the generalized Einstein coefficient ${k}_{E}$, and ${V}_{fmax}$—a maximum packing fraction of filler particles. The values of $A$ and ${V}_{fmax}$ for different shapes and arrangements of particles are given in the reference^45^. In the current study for spherical particles, $A$ = 1.5 and ${V}_{fmax}$ = 0.6 (body-centered cubic arrangement) are used in calculations.

The Agari model offers an alternative method for analyzing thermal conductivity^40,41^. This empirical model considers the dispersion state of the filler and the matrix structure by introducing two parameters, ${C}_{1}$ and ${C}_{2}$. The first coefficient, ${C}_{1}$, is related to the crystallinity and crystalline dimension of a polymer, and, the second one, ${C}_{2}$, is the free factor, which indicates the ability of forming a heat conductive network for fillers. Therefore, ${C}_{2}$ would significantly change with an increase of filler content in composites. The empirical Agari model, reflecting the actual structure of composites, effectively fits thermal conductivity data in polymer composites filled with both micro- and nanoparticles^42,46^.

**Figure 6:**
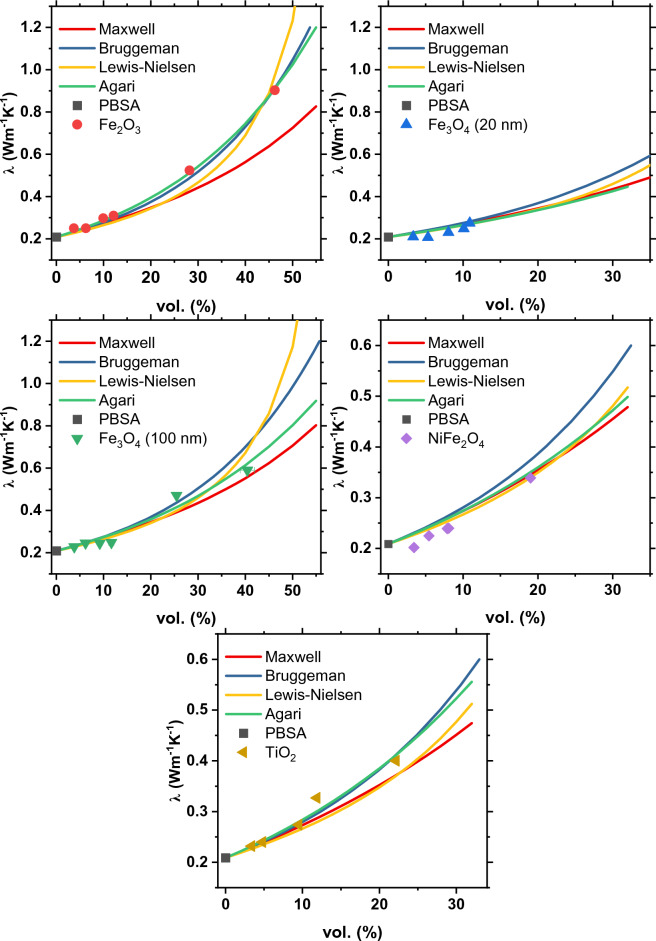
Thermal conductivities of composites at 25 °C and their approximation to mathematical models.

Figure 6 displays how the thermal conductivity data of PBSA composites align with the models listed in Table 3. The analysis uses a thermal conductivity value of 0.209 Wm^−1^ K^−1^ for the polymer matrix, and varying conductivities for fillers (in Wm^−1^ K^−1^): 8.4 (TiO_2_), 10 (NiFe_2_O_4_), 6 (Fe_2_O_3_), and 5 (Fe_3_O_4_). When approximating with the Agari model, it was assumed that the structure of PBSA is not affected by the presence of metal oxide particles, so ${C}_{1}$ is taken as unity. Best fits for the Agari model were obtained with the parameter ${C}_{2}$ equal to 0.7, 0.5, 0.9, 0.7, and 0.5 for composites filled with TiO_2_, NiFe_2_O_4_, Fe_2_O_3_, Fe_3_O_4_ (100 nm), and Fe_3_O_4_ (20 nm), respectively. According to the ${C}_{2}$ formulation, this parameter indicates the ease of creating thermally conductive filler networks specific to each polymer/filler system. The higher the ${C}_{2}$, the more efficient thermal conductivity appears. Following this line of reasoning, a composite containing Fe_2_O_3_ particles (${C}_{2}$ = 0.9) proves to be the most effective in enhancing thermal conductivity of PBSA.

A comparison of experimental thermal conductivity with the considered theoretical and empirical models shows that all approaches are able to reasonably predict the thermal conductivity of composites at low filler content and up to 20 vol.%. At higher filler loadings, the Bruggeman and Agari models provide better prediction due to their ability to account for interactions between fillers.

To determine how temperature affects thermal conductivity of the composites, we calculated the activation energies ${E}_{a}$ (Table 4 and Figure S12) from Arrhenius plots (Figure S11) of the thermal conductivity measurements at 25, 35, 45 °C. Thermal activation energy refers to the energy required to overcome a thermally activated potential barrier, i.e. the energy per molecule necessary for phonons or other thermal carriers to begin to participate in thermal conductivity. Adding 3–5 vol.% fillers to PBSA matrix doubled the temperature's impact on thermal conductivity, but this effect diminished with higher filler content. For Fe_2_O_3_ and Fe_3_O_4_, at maximum filler loadings, the temperature dependance decreased below that of the neat PBSA. The composites with 5 vol.% NiFe_2_O_4_ and 3 vol.% TiO_2_ exhibited the highest temperature dependency. The inherent thermal conductivity ${\uplambda }_{0}$ at infinite temperature (Table 4) inversely correlates with filler concentration for TiO_2_ and Fe_3_O_4_.

**Table 4:** The activation energies and the inherent thermal conductivity at infinite temperature.

Filler	Vol. (%)	$\left|{E}_{a}\right|$± Δ (eV)	${\uplambda }_{0}$± Δ (Wm^-1^ K^-1^)*
PBSA	0	0.048 ± 0.008	${1.37 }_{-\hspace{0.17em}\text{0.37 }}^{+0.50}$
TiO_2_ (5 nm)	3	0.097 ± 0.011	${9.88 }_{-\hspace{0.17em}\text{3.41 }}^{+5.21}$
	5	0.084 ± 0.016	${6.25 }_{-\hspace{0.17em}\text{2.85 }}^{+5.23}$
	10	0.069 ± 0.016	${4.04 }_{-\hspace{0.17em}\text{1.87 }}^{+3.47}$
	12	0.055 ± 0.006	${2.75 }_{-\hspace{0.17em}\text{0.56 }}^{+0.70}$
	22	0.053 ± 0.004	${3.20 }_{-\hspace{0.17em}\text{0.49 }}^{+0.58}$
NiFe_2_O_4_ (30 nm)	3	0.081 ± 0.003	${4.66 }_{-\hspace{0.17em}\text{0.48 }}^{+0.53}$
	5	0.099 ± 0.004	${10.74 }_{-\hspace{0.17em}\text{1.48 }}^{+1.72}$
	8	0.080 ± 0.012	${5.34 }_{-\hspace{0.17em}\text{1.92 }}^{+3.00}$
	8*	0.047 ± 0.012	${1.45 }_{-\hspace{0.17em}\text{0.54 }}^{+0.86}$
	19	0.073 ± 0.001	${5.75 }_{-\hspace{0.17em}\text{0.11 }}^{+0.11}$
Fe_2_O_3_ (100 nm)	4	0.072 ± 0.008	${4.04 }_{-\hspace{0.17em}\text{1.08 }}^{+1.47}$
	6	0.073 ± 0.006	${4.35 }_{-\hspace{0.17em}\text{0.94 }}^{+1.21}$
	10	0.067 ± 0.008	${4.00 }_{-\hspace{0.17em}\text{1.02 }}^{+1.36}$
	12	0.068 ± 0.002	${4.34 }_{-\hspace{0.17em}\text{0.38 }}^{+0.42}$
	28	0.031 ± 0.001	${1.74 }_{-\hspace{0.17em}\text{0.07 }}^{+0.07}$
	46	0.032 ± 0.002	${3.15 }_{-\hspace{0.17em}\text{0.26 }}^{+0.28}$
Fe_3_O_4_ (100 nm)	4	0.085 ± 0.002	${6.32 }_{-\hspace{0.17em}\text{0.42 }}^{+0.45}$
	6	0.074 ± 0.010	${4.49 }_{-\hspace{0.17em}\text{1.41 }}^{+2.07}$
	9	0.076 ± 0.005	${4.67 }_{-\hspace{0.17em}\text{0.75 }}^{+0.90}$
	12	0.065 ± 0.020	${3.07 }_{-\hspace{0.17em}\text{1.64 }}^{+3.52}$
	25	0.012 ± 0.001	${0.75 }_{-\hspace{0.17em}\text{0.01 }}^{+0.01}$
	40	0.039 ± 0.018	${2.62 }_{-\hspace{0.17em}\text{1.31 }}^{+2.63}$
Fe_3_O_4_ (20 nm)	3	0.071 ± 0.005	${3.38 }_{-\hspace{0.17em}\text{0.53 }}^{+0.63}$
	5	0.079 ± 0.001	${4.43 }_{-\hspace{0.17em}\text{0.22 }}^{+0.23}$
	8	0.059 ± 0.009	${2.28 }_{-\hspace{0.17em}\text{0.68 }}^{+0.98}$
	10	0.065 ± 0.020	${3.07 }_{-\hspace{0.17em}\text{1.64 }}^{+3.52}$
	11	0.048 ± 0.003	${1.76 }_{-\hspace{0.17em}\text{0.20 }}^{+0.22}$

*An asymmetric error range occurs due to linear mean-square-error approximation within semi-logarithmic coordinates.

### Dielectric properties

**Figure 7:**
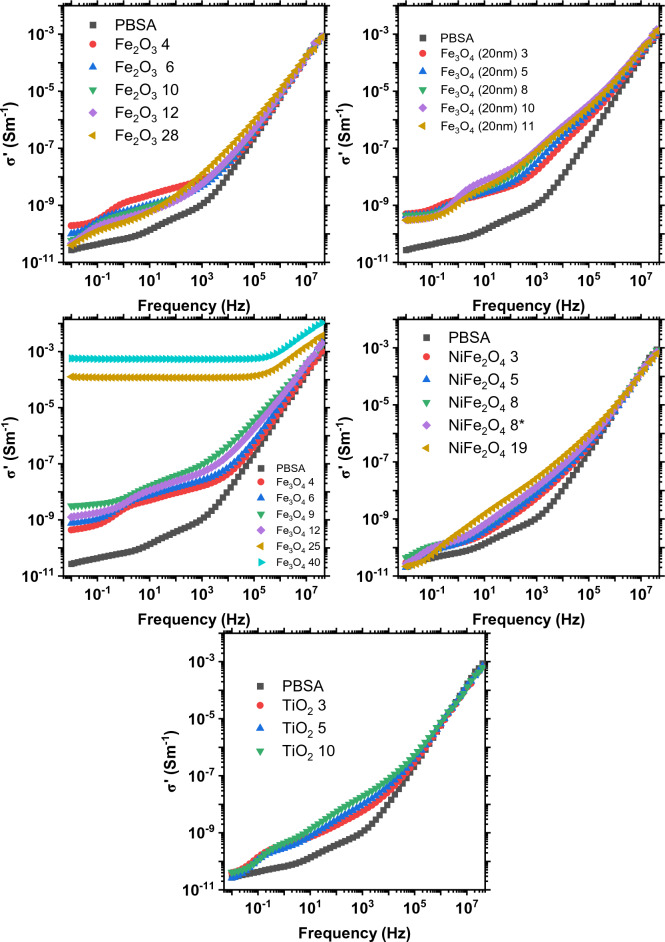
AC conductivity spectra of the composites.

**Figure 8:**
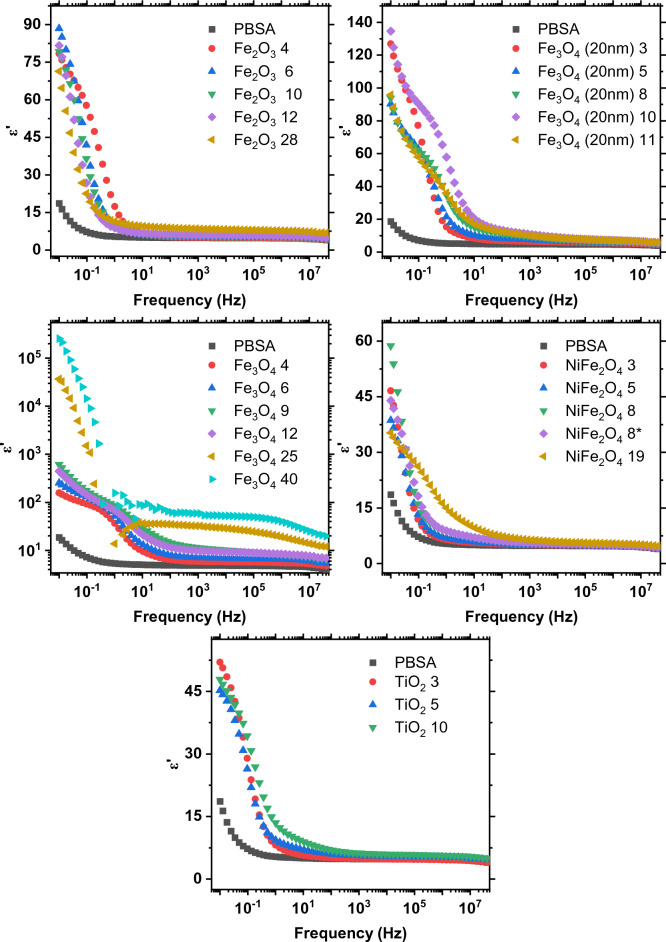
Complex dielectric permittivity spectra of the composites.

The AC conductivity and complex dielectric permittivity spectra of the composites were determined at frequencies ranging from 10^–2^ to 4 × 10^7^ Hz to establish their low-frequency electric-dielectric properties, as shown in Figs. 7 and 8. In electronics and power systems, composites are used to enhance low frequency signal handling. They aid in impedance matching, provide EMI shielding, and serve other related functions.

The incorporation of fillers enhanced the AC conductivity of the composites, as shown in Fig. 7. This enhancement varied depending on the type of filler used. However, only the two composites with the highest loading of 100 nm Fe_3_O_4_ exhibited a region of frequency independent conductivity. Fe_3_O_4_ and NiFe_2_O_4_ are spinel ferrites, however, only Fe_3_O_4_ is a conductor. β-TiO_2_^48^_,_ α-Fe_2_O_3_^49^, and NiFe_2_O_4_^50^ are semiconductors. The conductivity slightly increased in the range of 10^–1^ to 10^4^ Hz for TiO_2_ and NiFe_2_O_4_ composites. The observed increases in conductivity can be attributed to two factors: firstly, the addition of a more conductive phase, namely, the filler; and secondly, the presence of impurity metals and ions within the filler material, which function as charge carriers. Surprisingly, Fe_2_O_3_ filled composites showed highest increase in conductivity with the lowest filler loading, observable up to 10^3^ Hz. The addition of 3–4 vol.% of 100 nm and 20 nm Fe_3_O_4_ both showed a similar increase of conductivity. Fe_3_O_4_ (100 nm) 25 and 40 vol.% composites showed frequency independent conductivity of 1.17 × 10^–4^ and 5.37 × 10^–4^ S/m respectively, which switched to frequency dependence at around 0.3 MHz. This behavior is explained by the formation of a percolated network. For spherical nanofillers percolation happens when a sufficient packing factor is reached to permit the electrical contact of particles throughout the composite. Gurland has estimated that the onset of percolation starts at approximately 30–40 vol.% of conductive filler^51^. This somewhat aligns with our results, but the difference could be attributed to filler shape and size, dispersion conditions, and the properties of the matrix^52^. The ferrimagnetic nature of Fe_3_O_4_ leads it to magnetically overpower dispersion forces, resulting in agglomeration. Consequently, this forms clusters that likely behave as larger, anisodiametric inclusions, thereby lowering the percolation threshold. The frequency independence at a certain critical frequency is a manifestation of the universal dielectric response and Jonscher’s law^53^.

The frequency-dependent permittivity of heterogeneous composites (Fig. 8) is largely determined by two key phenomena. The first is the Maxwell–Wagner-Sillars^54^ effect, describing space charge polarization in dielectric composites. The second is Koops theory^55^, which explains semiconductor-filled composites as a system of conductive grains divided by resistive grain boundaries, focusing on the accumulation of charge at these boundaries. The high dielectric permittivity values in the Fe_3_O_4_ composites at low frequencies could be attributed to space charge polarization. Notably, different authors have described this phenomena in other ferrite materials such as Zn–Cu ferrites^56^ and Li–Ni–Zn ferrites^57^. Fe_3_O_4_ composites also exhibit increased dielectric permittivity due to charge hopping between the Fe^+2^ and Fe^+3^ ions at lower frequencies as described in our previous paper^14^. With the presence of charge carriers this phenomenon is intensified at the highest concentrations, where conductive paths are established throughout the entire composite. In other metal oxide composites, space charge polarization occurs at lower frequencies. Above the relaxation frequencies of these space charges, the dielectric response becomes uniform and frequency-independent, transitioning to a state dominated by the matrix. In this state, the response is primarily controlled by the relaxation processes of the polymer^58^.

### EMI shielding properties

To assess the potential of these composites for EMI shielding applications, we evaluated their shielding efficiency (SE) across various frequencies (Table 5). This was done using Eqs. (5)–(7), which consider losses due to both dielectric and conductive effects. The SE decreases as the field frequency increases. SE values were notably high at 0.01 Hz, reaching 77 dB for the 25 vol.% and 84 dB for the 40 vol.% Fe_3_O_4_ (100 nm) loadings. However, as the frequency increased, there was a significant decrease in SE values, dropping to 9 dB and 11 dB for 25 vol.% and 40 vol.% composites, respectively. The high SE values of Fe_3_O_4_ (100 nm) are caused by the formation of a conductive particle network. This network enhances electrical conductivity and dielectric losses, reflecting or effectively dissipating the EM field as heat. Fe_2_O_3_ showed the second highest SE at the lowest frequencies. Notably, these SE values were inversely proportional to the filler concentration up to 10 Hz. This trend is associated with the dielectric relaxation processes that were discussed previously. The 4 vol.% Fe_2_O_3_ composite is notable as it provides adequate EM shielding at a very low filler content, while simultaneously maintaining its non-conductive nature. It is important to evaluate the SE across different parts of the EM spectrum, as it varies depending on the specific application. Commonly, EMI in the very-low frequency or audio frequency, from direct-current non-alternating to 20 kHz alternating (shaded blue in Table 5), is generated by analog systems, electrical devices, power transmission equipment and others. This type of EMI is typically produced through conduction. Radio frequency interference is defined as EMI at frequencies from 20 kHz to optical wavelengths (shaded orange in Table 5). It is caused by radiative sources, such as wireless and radio communications systems, processors and microcontrollers, and high frequency equipment^59,60^.

**Table 5:** Calculated EMI SE of the composite materials.

Filler	vol (%)	SE at frequency (dB)									
		0.01 Hz	0.1 Hz	1.0 Hz	10.6 Hz	112 Hz	0.84 kHz	8.89 kHz	93.70 kHz	0.99 MHz	10.41 MHz
PBSA	–	13.02	8.28	6.73	6.54	6.47	6.46	6.45	6.43	6.39	6.27
TiO_2_ (5 nm)	3	14.99	12.14	8.23	6.85	6.55	6.46	6.42	6.39	6.35	6.22
	5	13.90	11.69	8.34	7.36	6.95	6.81	6.74	6.70	6.65	6.52
	10	14.97	12.32	9.40	8.01	7.32	7.06	6.95	6.89	6.83	6.72
NiFe_2_O_4_ (30 nm)	3	14.19	10.01	7.24	6.80	6.64	6.57	6.52	6.48	6.43	6.30
	5	13.21	9.92	7.41	6.90	6.66	6.56	6.49	6.44	6.39	6.26
	8	15.36	10.90	8.04	7.37	7.02	6.86	6.76	6.69	6.62	6.48
	8*	13.93	10.80	8.06	7.36	6.96	6.79	6.68	6.61	6.54	6.40
	19	13.16	11.22	9.53	8.21	7.43	7.12	6.92	6.79	6.70	6.56
Fe_2_O_3_ (100 nm)	4	20.20	14.72	10.99	7.51	6.62	6.51	6.47	6.44	6.40	6.27
	6	17.97	13.89	9.05	7.04	6.72	6.67	6.64	6.61	6.56	6.42
	10	16.44	13.22	8.84	7.19	6.90	6.84	6.80	6.76	6.70	6.57
	12	16.02	12.45	8.34	7.21	7.02	6.96	6.91	6.87	6.81	6.68
	28	15.49	11.57	8.74	8.10	7.89	7.79	7.71	7.64	7.55	7.41
Fe_3_O_4_ (100 nm)	4	23.47	17.07	13.40	8.76	7.12	6.89	6.84	6.80	6.75	6.60
	6	25.75	18.72	14.62	9.78	7.69	7.36	7.28	7.23	7.16	6.97
	9	31.68	23.07	16.56	12.32	9.43	8.65	8.30	8.04	7.76	7.46
	12	27.89	20.34	15.29	11.15	8.71	8.22	8.06	7.93	7.78	7.54
	25	77.49	67.48	57.21	46.99	36.82	28.21	18.62	11.85	10.18	9.16
	40	84.07	74.08	63.81	53.58	43.37	34.67	24.71	15.96	12.67	11.00
Fe_3_O_4_ (20 nm)	3	23.97	17.08	11.40	7.99	7.29	7.15	7.00	6.86	6.75	6.58
	5	23.49	16.07	11.69	8.50	7.76	7.54	7.27	7.05	6.90	6.69
	8	23.53	15.88	12.66	9.61	8.63	8.26	7.78	7.42	7.20	6.95
	10	22.11	16.05	14.10	10.67	9.06	8.53	7.99	7.62	7.38	7.11
	11	21.91	15.17	12.46	9.94	8.84	8.33	7.86	7.57	7.37	7.13

**Figure 9:**
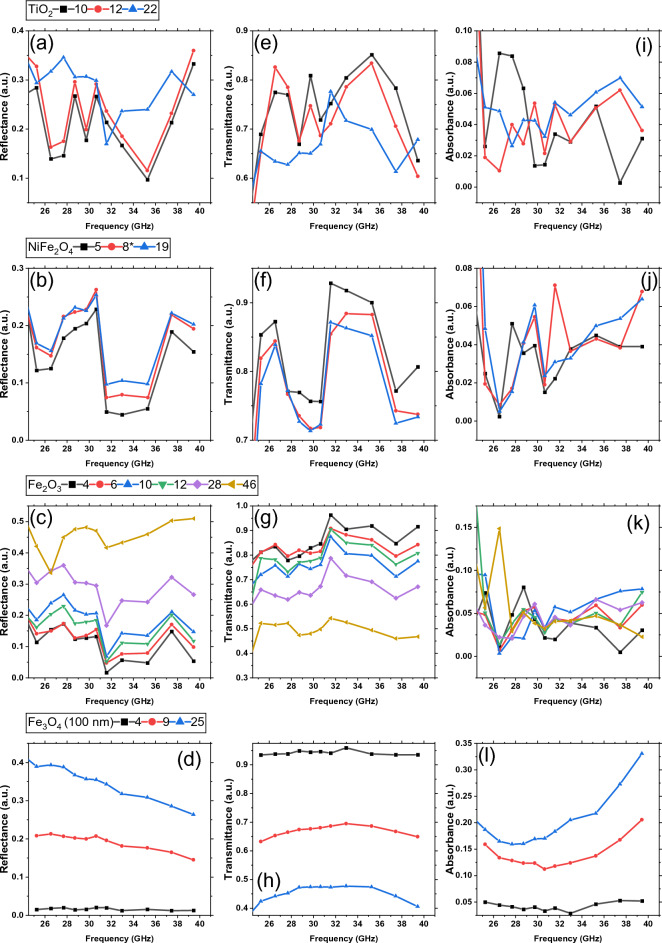
Composite reflectance (**a**–**d**), transmittance (**e**–**h**), and absorbance (**i**–**l**) spectra at 25–40 GHz.

To further assess the EM shielding efficacy of the composites, we conducted additional reflectance, transmission, and absorption measurements across microwave frequencies (25–40 GHz) as depicted in Fig. 9. Composites containing TiO_2_ (Figs. 9a, e, i) and NiFe_2_O_4_ (Fig. 9b, f, j) exhibited exceptionally low absorbance values, approaching the sensitivity limits of our measurement apparatus. Notably, the reflectance and transmittance values varied with filler concentration, with the 22 vol.% TiO_2_ composite achieving a minimal transmittance of 0.61 at 37.5 GHz. In contrast, composites filled with Fe_2_O_3_ and Fe_3_O_4_ (100 nm) showed much more uniform reflectance spectra, where reflectance increases with filler concentration. From the Fe_2_O_3_ composites (Figs. 9c, g, k), only the 46 vol.% sample showed a clear frequency dependence, with reflectance increasing to a maximum of 0.51 at 39.5 GHz. This sample also showed a transmission minimum of 0.47 at the same frequency. Composites filled with Fe_3_O_4_ (100 nm) emerged as superior EM absorbers, particularly the 25 vol.% composite which exhibited a maximal absorbance of 0.33 at 39.5 GHz (Fig. 9l) and consistently the lowest broadband transmittance values between 0.40 and 0.48 (Fig. 9h). Figure 8d illustrates a decrease in reflectance with rising frequency, attributable to the skin effect in conductive materials.

Our findings underscore the potential of these composites as cost-effective EMI shielding additives across the low audio frequency range, as well as EM reflectors (Fe_2_O_3_, TiO_2_) or absorbers (Fe_3_O_4_) at higher frequencies. However, it is important to note that this effect should be considered supplementary to the other properties of the composites. Future research should explore different frequency bands and incorporate considerations of the magnetic permeabilities of the composites to expand their applicability in EM shielding applications.

## Conclusions

In this paper, we incorporated TiO_2_ (5 nm), NiFe_2_O_4_ (30 nm), Fe_2_O_3_ (100 nm), and Fe_3_O_4_ (20 nm and 100 nm) fillers in a PBSA matrix via a solvent casting process to create nanocomposites with enhanced thermal conductivity and dielectric properties. The solvent mixing process showed a variable compatibility of nanofillers with the PBSA matrix, which limited the maximum filler loading. SEM–EDX element mapping revealed that the examined composites had an uniform distribution of well dispersed nanoparticles, exhibiting no signs of agglomeration or major structural defects.

The LFA data were fitted to various theoretical and empirical models (Maxwell, Hatta, Bruggeman, Lewis-Nielsen, Agari). We show that the models are able to reasonably predict the thermal conductivity of the composites. At higher filler contents (above 20 vol.%) Bruggeman and Agari provide better fits, due to increased interaction between filler particles. Out of the 28 prepared composites, the Fe_2_O_3_ 46 vol.% filled composite reached the highest thermal conductivity values (0.9 Wm^−1^ K^−1^ at 25 °C), while also remaining electrically insulating. It is evident, that the maximum thermal conductivity values are more limited by the packing and interaction of fillers, rather than their intrinsic conductivities.

BDS showed that the composites with 25 and 46 vol.% Fe_3_O_4_ (100 nm) were the only compositions that achieved percolation thresholds and formed conductive networks, while other tested compositions preserved their insulating properties. The 46 vol.% Fe_3_O_4_ (100 nm) composite shows good EM shielding in the 10^–2^–4 × 10^4^ Hz region, that drops from 85 to 25 dB with increasing frequency, while the 25 vol.% composite achieved only slightly lower results. Other selected metal oxide fillers showed weak EM shielding efficiency in the tested frequency range. In the microwave range (25–40 GHz), all composites showed a variable reflectance depending on the filler concentration and frequency, with 46 vol.% Fe_2_O_3_ achieving the highest reflectance (~ 0.5). The overall best shielding effectiveness was attained in the Fe_3_O_4_ containing composites, which also had the highest absorbance values (0.2–0.32) at 25 vol.%.

The investigated nanocomposites can be used as relatively adjustable, cheap, eco-friendly, and electrically insulating thermally conductive materials for thermal management and heat dissipation.

### Supplementary Information

Supplementary Information.

## Data Availability

The data presented in this study are available on request from the corresponding author.
