# Supplementary material for: Evaluation of thermal conductivity models and dielectric properties in metal oxide-filled poly(butylene succinate-co-adipate) composites
Source: Sci Rep. 2024 Jun 13;14:13629. doi: 10.1038/s41598-024-64426-5 (PMC11176320; doi:10.1038/s41598-024-64426-5)
Supplement: Supplementary file 1 — Supplementary Information. [file 41598_2024_64426_MOESM1_ESM.docx]

Evaluation of thermal conductivity models and dielectric properties in metal oxide-filled poly(butylene succinate-co-adipate) composites

Miks Bleija^1,*^, Oskars Platnieks^1^, Olesja Starkova^2^, Jan Macutkevič^3^, Dzmitry Tsyhanok^3^, Liga Orlova^4^, and Sergejs Gaidukovs^1^

^1^Institute of Chemistry and Chemical Technology, Faculty of Natural Sciences and Technology, Riga Technical University, P. Valdena 3, LV-1048 Riga, Latvia
^2^Institute for Mechanics of Materials, University of Latvia, Jelgavas 3, LV-1004 Riga, Latvia
^3^Faculty of Physics, Vilnius University, Sauletekio 9, LT-10222 Vilnius, Lithuania
^4^Institute of Materials and Surface Engineering, Faculty of Natural Sciences and Technology, Riga Technical University, P. Valdena 3, LV-1048 Riga, Latvia
^*^miks.bleija@rtu.lv

Supplementary Information


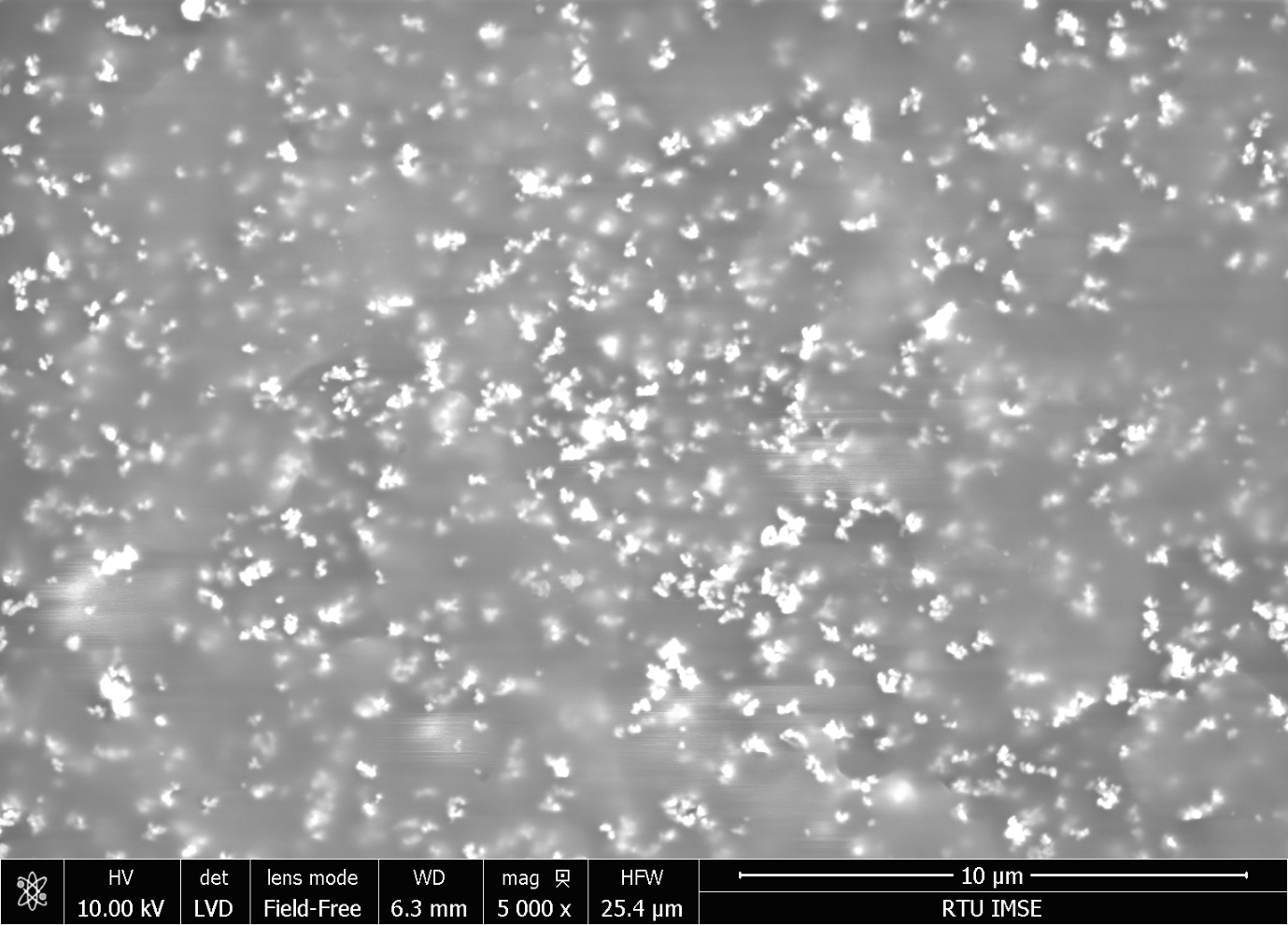
Figure S1. 4 vol.% Fe_2_O_3_ composite at 5 000 × magnification.


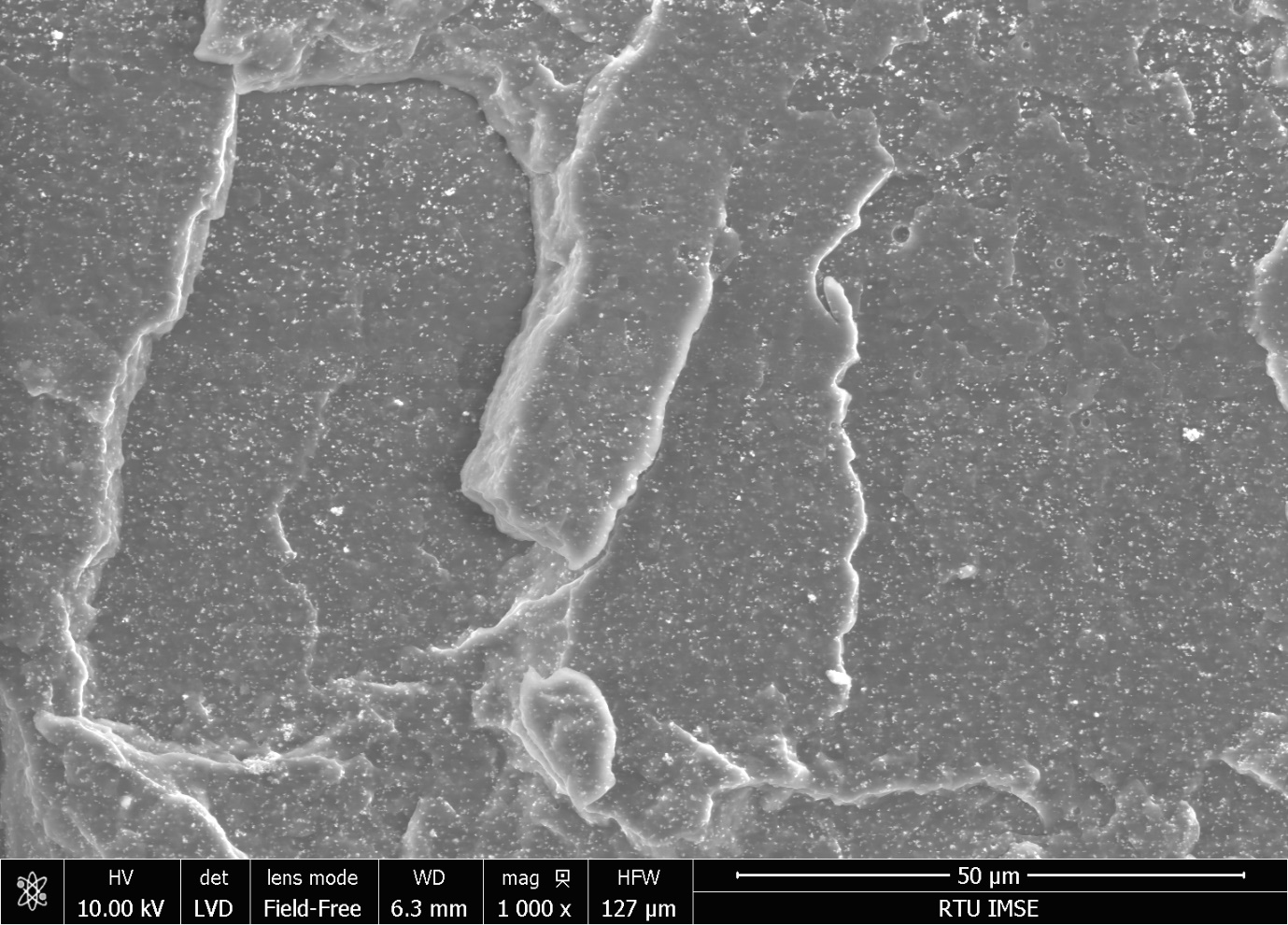
Figure S2. 4 vol.% Fe_2_O_3_ composite at 1 000 × magnification.


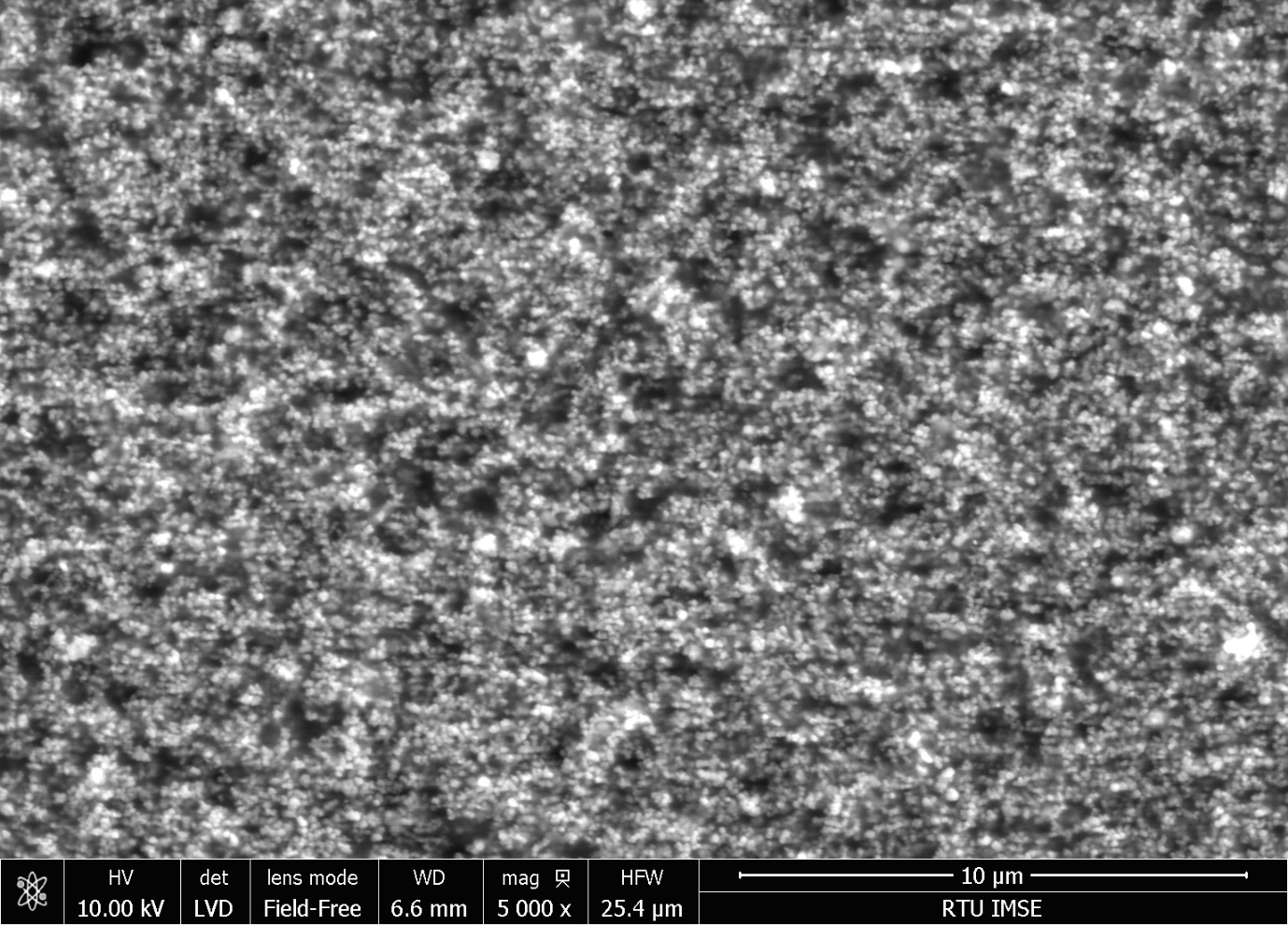
Figure S3. 28 vol.% Fe_2_O_3_ composite at 5 000 × magnification.


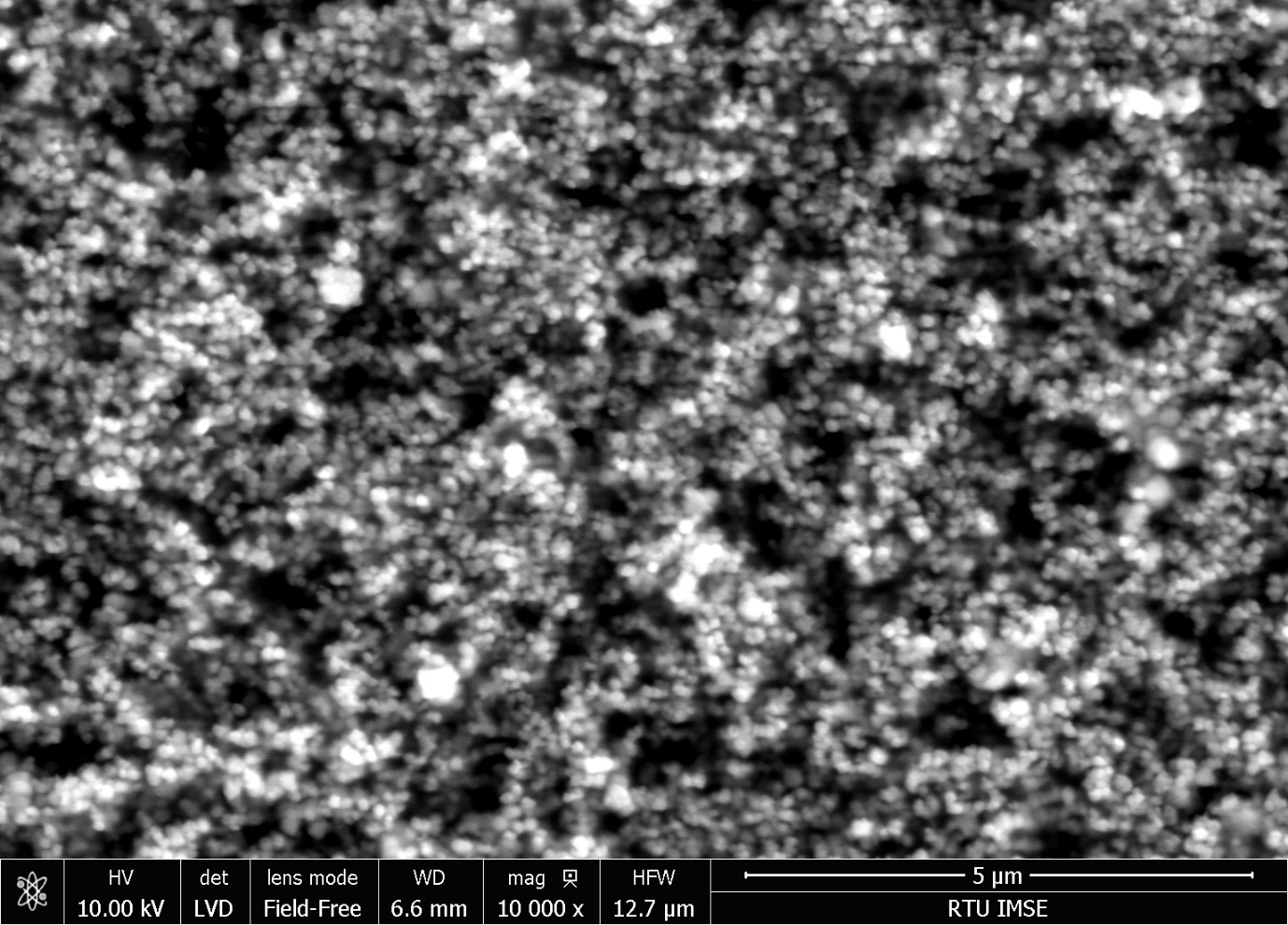
 Figure S4. 28 vol.% Fe_2_O_3_ composite at 10 000 × magnification.


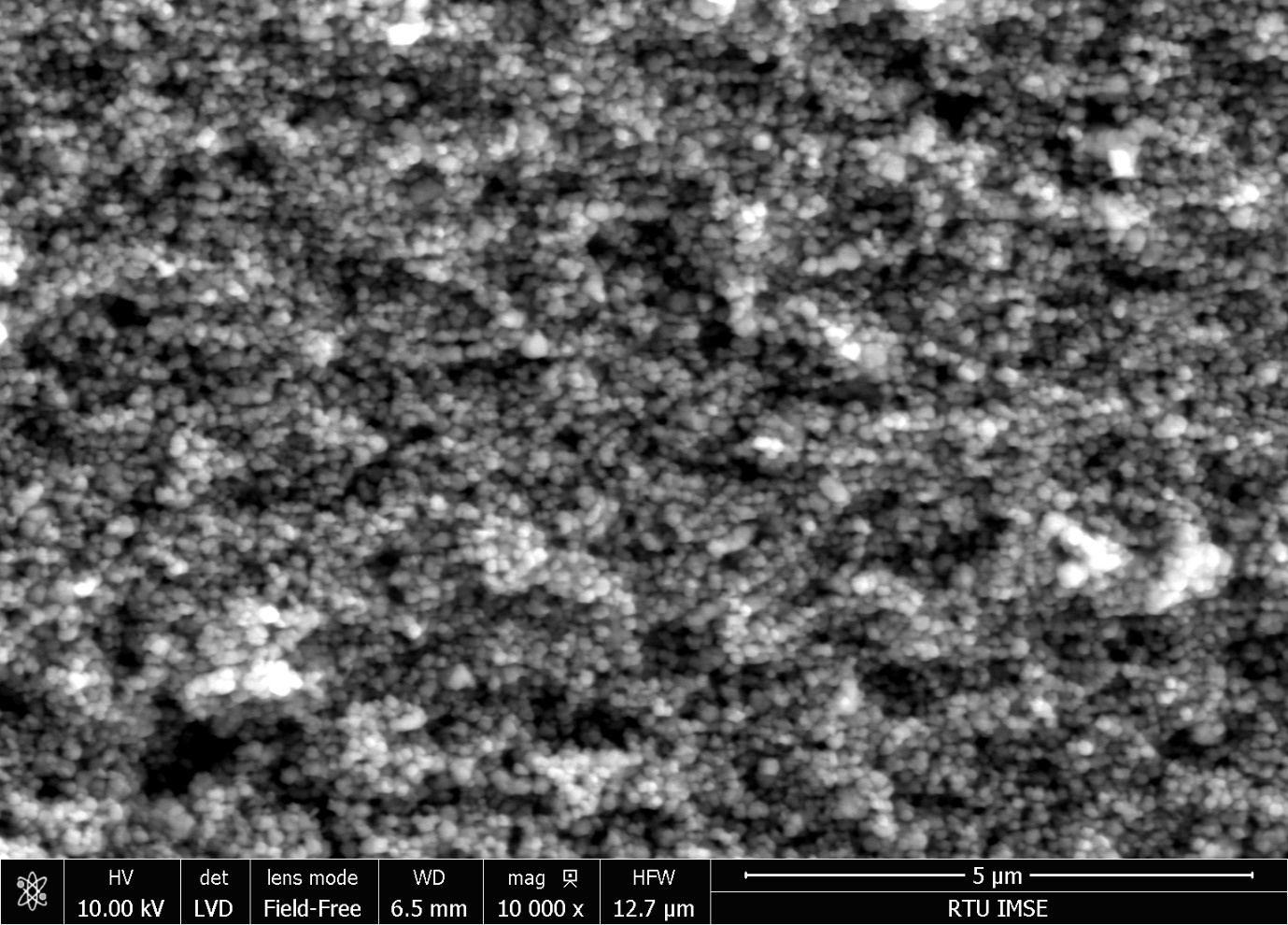
Figure S5. 46 vol.% Fe_2_O_3_ composite at 10 000 × magnification


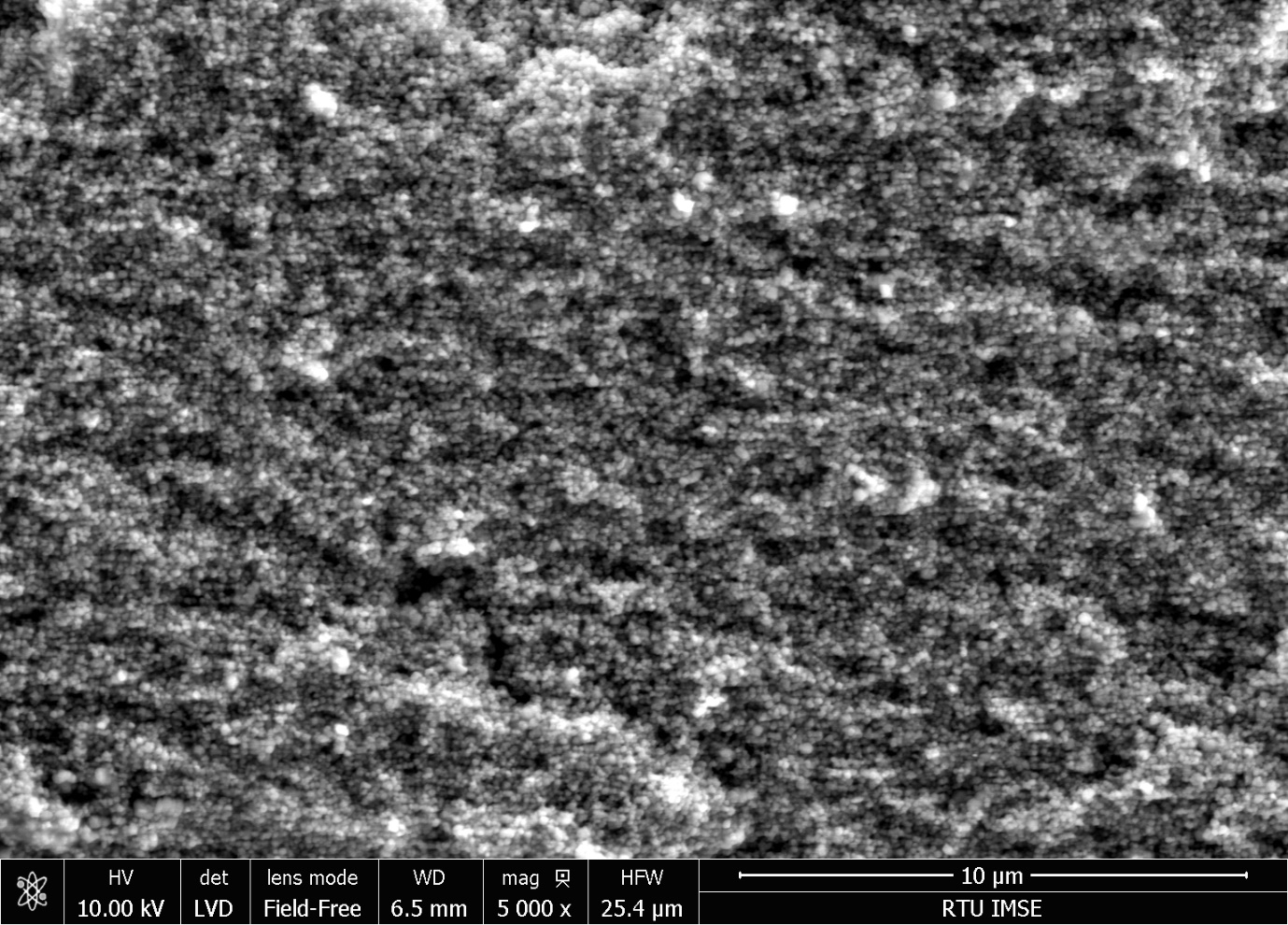
 Figure S6. 46 vol.% Fe_2_O_3_ composite at 5 000 × magnification.


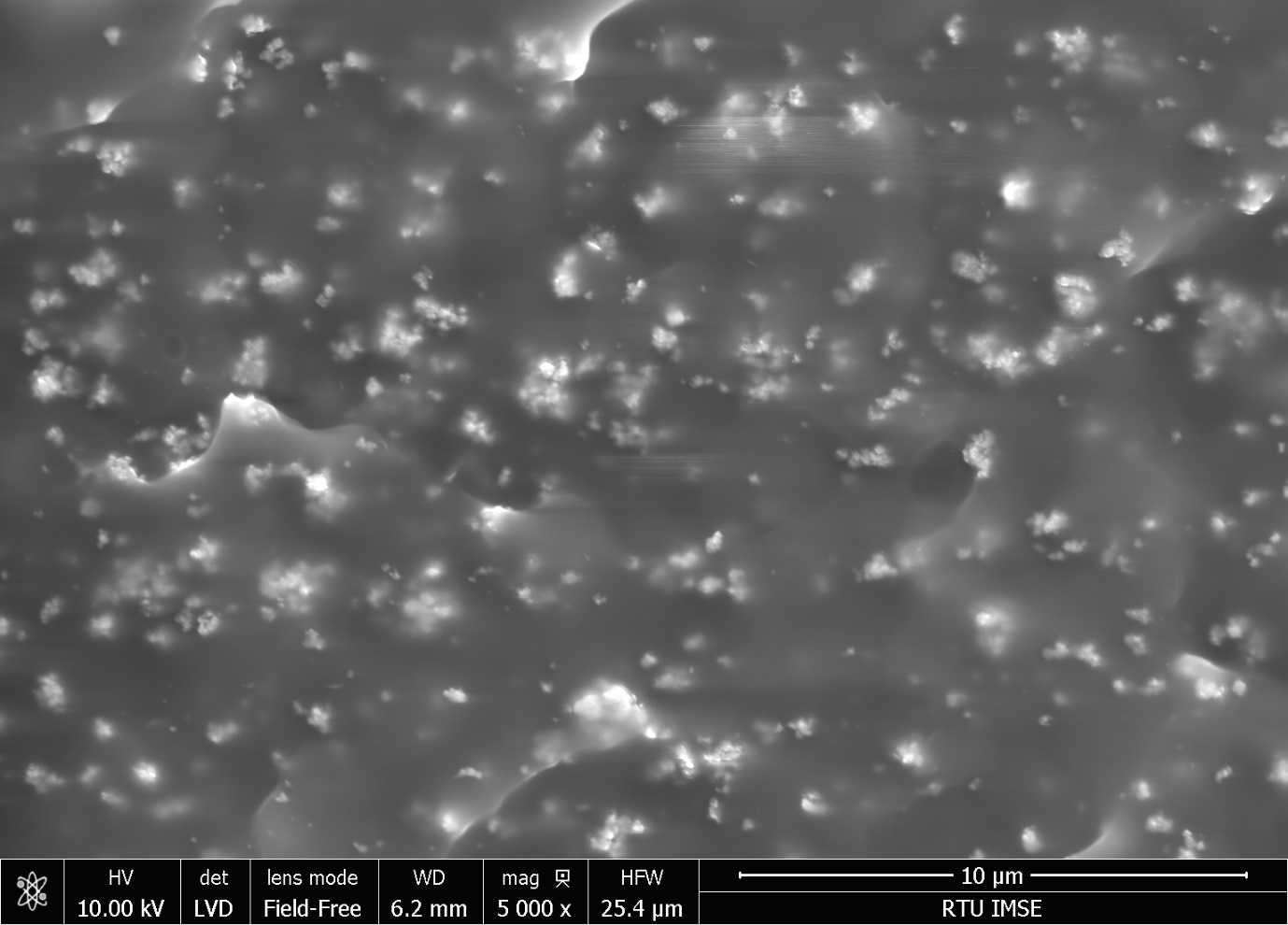
Figure S7. 4 vol.% Fe_3_O_4_ (100 nm) composite at 5 000 × magnification.


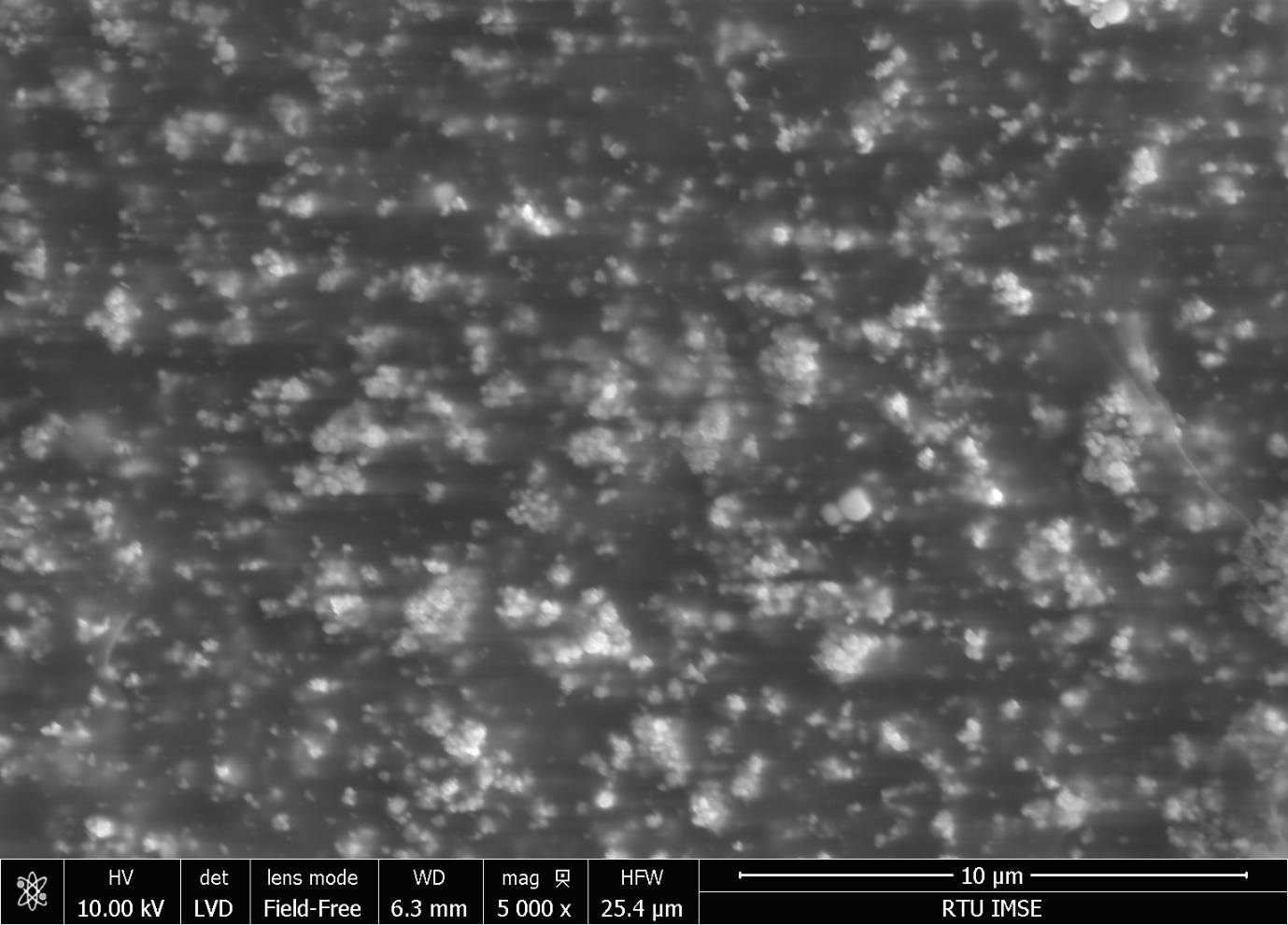
Figure S8. 25 vol.% Fe_3_O_4_ (100 nm) composite at 5 000 × magnification.


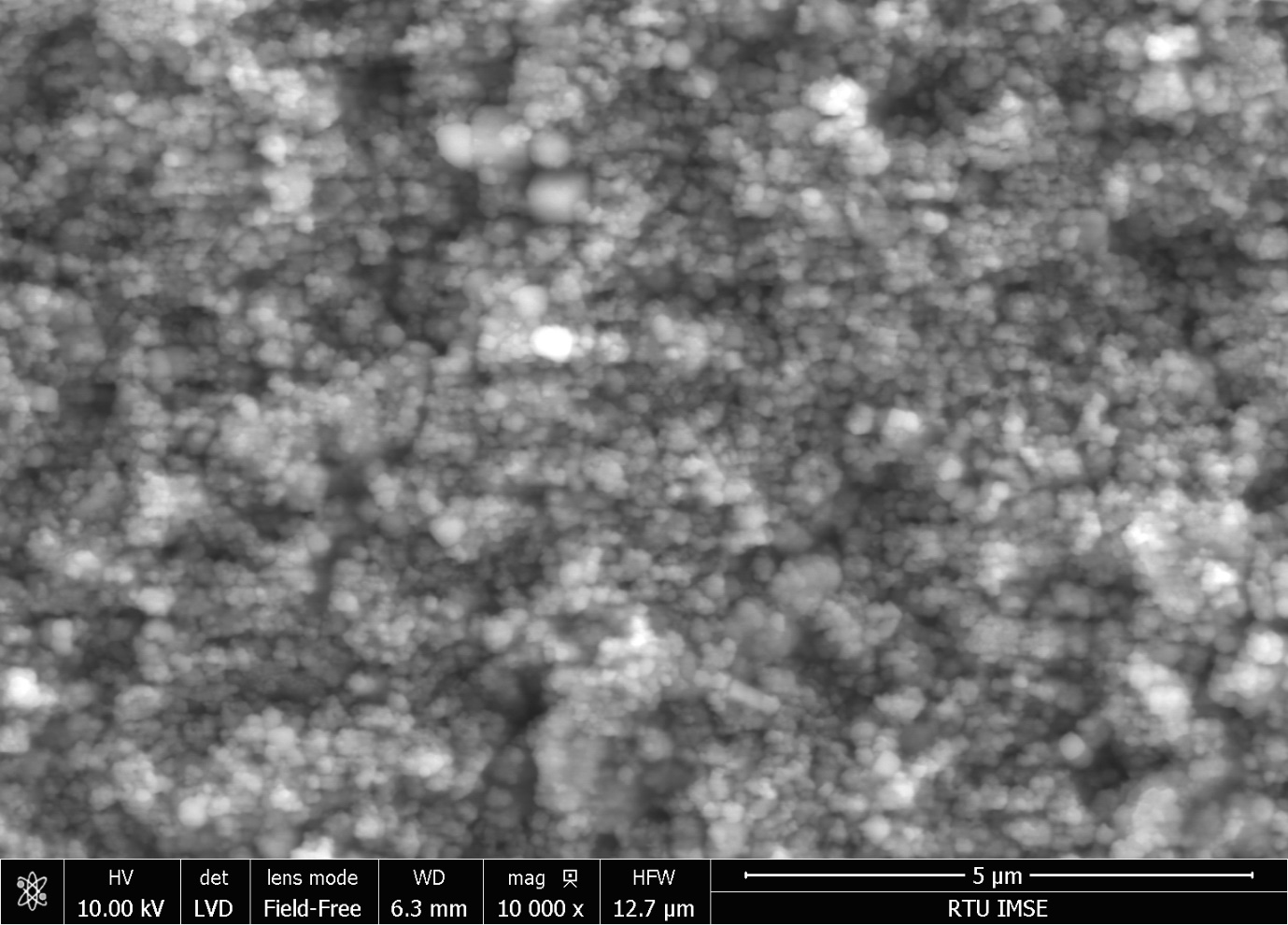
Figure S9. 40 vol.% Fe_3_O_4_ (100 nm) composite at 10 000 × magnification.


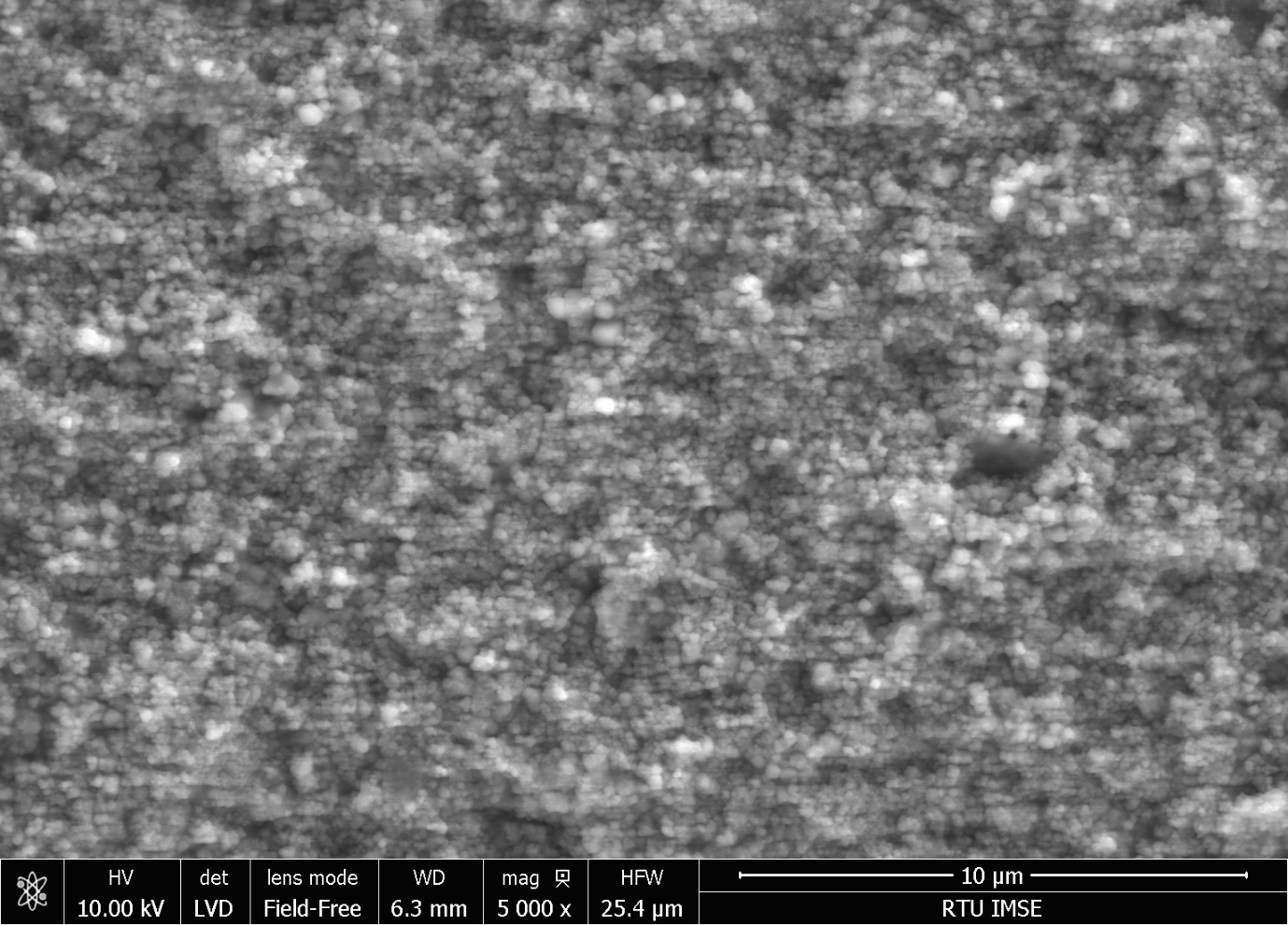
Figure S10. 40 vol.% Fe_3_O_4_ (100 nm) composite at 5 000 × magnification.

Figure S11. Arrhenius plots of the thermal conductivity measurements at 25, 35, 45 °C

Figure S12. Activation energies of thermal conductivity at 25-45 °C
